# Reactive sulfur species are inactivated and excreted as trimethylsulfonium ion by thiopurine *S*-methyltransferase

**DOI:** 10.1016/j.redox.2026.104144

**Published:** 2026-04-01

**Authors:** Yasunori Fukumoto, Momoka Uchida, Yoshikazu Yamagishi, Natsu Saito, Ayune Watanabe, Rina Mukasa, Ryosuke Kobayashi, Takuro Horii, Izuho Hatada, Yu-ki Tanaka, Noriyuki Suzuki, Yasumitsu Ogra

**Affiliations:** a Graduate School of Pharmaceutical Sciences, Chiba University, Chiba, 260-8675, Japan; b Graduate School of Medicine, Chiba University, Chiba, 260-8670, Japan; c Laboratory of Genome Science, Biosignal Genome Resource Center, Institute for Molecular and Cellular Regulation, Gunma University, Gunma, 371-8512, Japan; d Laboratory of Veterinary Anatomy, School of Veterinary Medicine, Rakuno Gakuen University, Hokkaido, 069-8501, Japan; e Viral Vector Core, Gunma University Initiative for Advanced Research (GIAR), Maebashi, 371-8511, Japan

**Keywords:** Reactive sulfur species, Sulfur methylation, Selenium metabolism, Methyltransferase, Thiol redox chemistry

## Abstract

Reactive sulfur species (RSS) play essential roles in cellular signaling, redox regulation, and defense against electrophilic stress. However, excessive RSS are cytotoxic and must be strictly regulated. While RSS elimination has been canonically attributed to oxidative pathways, here we show that thiopurine *S*-methyltransferase (TPMT), a selenium-associated methyltransferase, promotes the detoxification and urinary excretion of RSS. In a mouse model, knockout of *TPMT* decreased urinary excretion of the trimethylsulfonium, suggesting that TPMT catalyzes a non-oxidative elimination of RSS via methylation. Cell-based assays demonstrated that TPMT exerts a protective effect against excess RSS. *In vitro*, TPMT methylated hydrosulfide, polysulfides (HSS^−^, HS_4_^−^), and persulfides (GSS^−^). ESI-MS analysis confirmed the formation of methylated GSS^−^. Kinetic analyses revealed substrate-dependent differences in methylation efficiency. *In silico* analyses suggested that steric hindrance of the substrate is a key determinant of the methylation efficiency. Collectively, these findings uncover an energy-dependent, non-oxidative pathway for the elimination of RSS beyond classical oxidative detoxification. Given that TPMT polymorphisms are clinically important determinants of thiopurine drug metabolism, variation in TPMT activity may likewise influence biological responses to RSS. Together, these results provide a foundation for understanding how endogenous methylation shapes RSS homeostasis.

## Highlights

• RSS undergo methylation-dependent inactivation followed by urinary excretion.
• TPMT was identified as the enzyme mediating RSS methylation and urinary excretion.
• TPMT-dependent methylation and mitochondrial oxidation form a dual-pathway model.
• Biochemical and *in silico* analyses revealed the molecular basis of RSS methylation.
• TPMT-dependent methylation suppresses RSS toxicity in cellular redox systems.

## Graphical abstract

**Figure:**
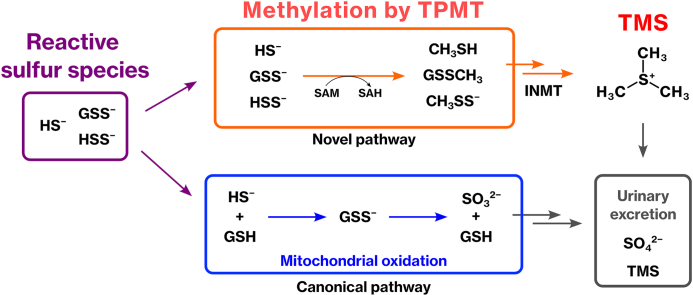

## 1 Introduction

Reactive sulfur species (RSS), such as hydrogen sulfide (H_2_S), persulfides, and polysulfides, play crucial roles in cellular signaling, redox regulation, and protection against electrophilic stress [[1], [2], [3]]. These sulfur-containing molecules act as antioxidants by scavenging reactive oxygen species and neutralizing electrophilic compounds, thereby maintaining redox homeostasis. Among RSS, H_2_S is well recognized as a gaseous signaling molecule with diverse physiological functions. However, accumulating evidence indicates that excess RSS can be cytotoxic, highlighting the importance of strictly regulating their intracellular levels. This dual nature, protective at physiological concentrations but harmful when accumulated, necessitates a balanced system for both RSS production and elimination.

The biosynthetic pathways of RSS have been well characterized, including the enzymatic generation of H_2_S via transsulfuration and the formation of persulfides and polysulfides through sulfur interconversion [1,2,4]. However, the mechanisms responsible for RSS clearance remain incompletely understood. Oxidative pathways are considered the primary route for RSS elimination. Such pathways involve mitochondrial enzymes, such as sulfide quinone oxidoreductase (SQOR) and persulfide dioxygenase (ETHE1) [2,5]. However, oxidative clearance alone may be insufficient under conditions of excess RSS. In such cases, available oxidants or electron acceptors become depleted. Moreover, the oxidative elimination of RSS risks disrupting the cellular redox equilibrium. These limitations underscore the need for alternative clearance mechanisms to process excess RSS safely. In this context, energy-dependent, non-oxidative pathways may serve as critical compensatory systems, particularly under reductive or high-RSS conditions. Nevertheless, such pathways have yet to be identified.

Selenium (Se), a chalcogen element closely related to sulfur in physicochemical properties, offers insights into potential alternative pathways for RSS metabolism. Certain RSS, such as persulfides and hydrosulfide (HS^−^), exhibit acid–base properties similar to those of Se-containing compounds, including comparable p*K*_a_ values and nucleophilicity [[6], [7], [8], [9]]. We recently demonstrated that reactive selenide species, likely present as selenide anions (HSe^−^ and glutathione-conjugated, GSSe^−^), are sequentially methylated by thiopurine *S*-methyltransferase (TPMT) and indolethylamine *N*-methyltransferase (INMT), forming excretable metabolite trimethylselenonium (TMSe). TPMT, originally characterized for methylating the thiol group of 6-mercaptopurine (6-MP), also acts on Se species [[10], [11], [12], [13]]. These observations led us to hypothesize that TPMT may similarly mediate the methylation of RSS, providing a non-oxidative pathway for RSS detoxification.

Here, we investigated whether TPMT catalyzes the methylation of RSS, including HS^−^, persulfides, and polysulfides. We employed a mouse model, cellular and biochemical assays, mass spectrometry, and *in silico* modeling to clarify physiological relevance, substrate specificity, and structural basis of TPMT-mediated RSS methylation. These efforts aimed to explore a non-oxidative, *S*-adenosylmethionine (SAM)-dependent pathway for RSS processing, thereby expanding our understanding of sulfur metabolism beyond classic oxidative mechanisms.

## 2 Results

### 2.1 *TPMT* is required for urinary excretion of trimethylsulfonium ion in mice

**Fig. 1:**
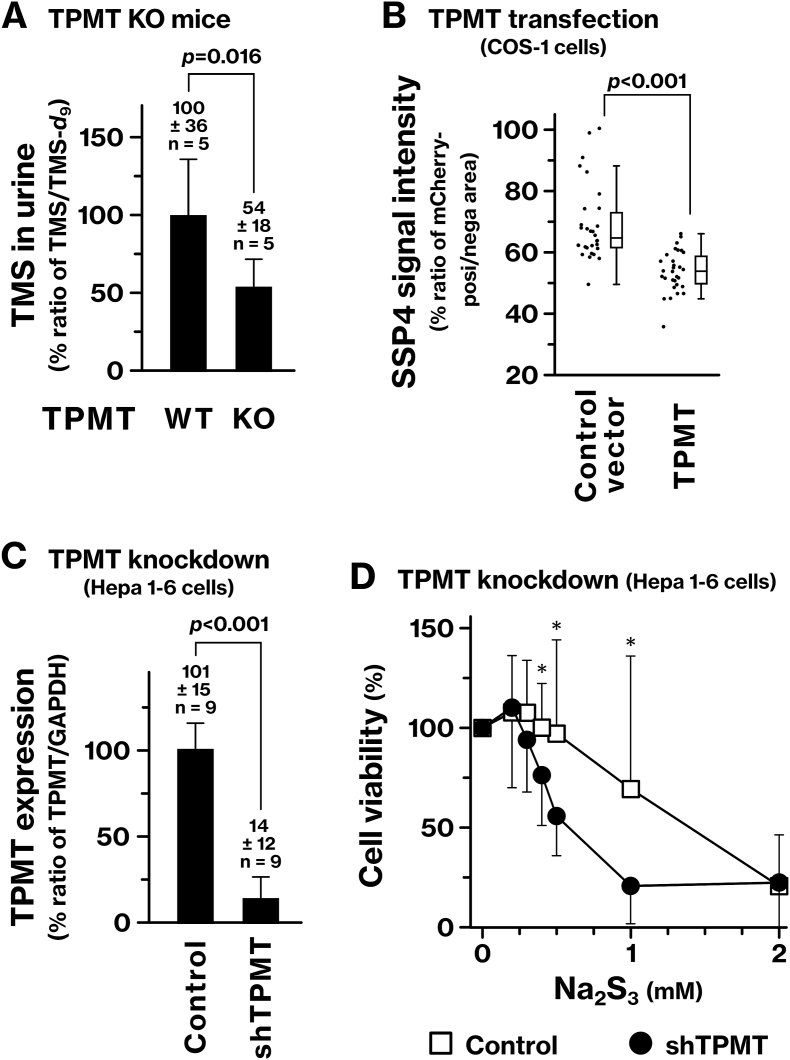
TPMT promotes urinary excretion and detoxification of RSS. **(A)** Trimethylsulfonium (TMS) in mouse urine was quantified by ESI-MS. Signal intensities were normalized to a deuterium-labeled TMS internal standard (TMS-*d*_9_). TPMT^−/−^ (TPMT-KO) mice were compared with parental BDF1 wild-type controls. **(B)** The effect of exogenous TPMT expression on endogenous RSS was examined. TPMT was transfected into COS-1 cells together with mCherry, and endogenous RSS were visualized using SSP4 staining. The SSP4 signal was quantified by fluorescence microscopy, and the ratio of the signal intensity in the mCherry-positive area to that in the mCherry-negative area was calculated for each microscopic image. For cells transfected with either TPMT or a control vector, 30 images were collected from three independent experiments and presented as a dot plot. Statistical significance was determined using Welch's *t*-test. **(C, D)***TPMT* was inhibited in Hepa 1-6 cells using short hairpin RNA (shRNA), and the knockdown was evaluated using quantitative PCR (C). The cells were exposed to Na_2_S_3_ for 24 h, and cell viability was determined using the MTS assay (D). *p*-values were calculated using Student's or Welch's *t*-test. *, *p* < 0.05.

Urinary excretion of trimethylsulfonium ion (TMS) has been previously reported [[14], [15], [16]], and in our ESI-MS analyses, TMS was readily detected in mouse urine. To evaluate the physiological role of TPMT in RSS methylation, we generated TPMT^−/−^ mice and quantified urinary TMS level. TPMT^−/−^ mice were viable to adulthood without evident growth retardation. Relative to wild-type controls, urinary TMS was significantly decreased in TPMT^−/−^ mice (Fig. 1A). These results raise the possibility that TPMT-mediated methylation is involved in the urinary excretion of RSS as TMS.

### 2.2 TPMT suppresses intracellular RSS accumulation and protects against RSS-induced cytotoxicity

To assess the role of TPMT in intracellular RSS regulation, we examined the effect of TPMT overexpression on endogenous RSS levels using the fluorescent probe SSP4. TPMT overexpression significantly reduced SSP4 fluorescence intensity in COS-1 cells (Fig. 1B), indicating that TPMT possesses sufficient activity for methylating and inactivating RSS *in vivo*.

Next, we evaluated whether endogenous TPMT mitigates the cytotoxic effects of excess RSS. TPMT knockdown by shRNA significantly decreased cell viability following exposure of Hepa 1-6 cells to trisulfide Na_2_S_3_ (Fig. 1C and D). These findings suggest that TPMT plays a protective role under elevated RSS conditions by reducing RSS cytotoxicity through methylation-dependent inactivation.

### 2.3 TPMT catalyzes disulfide methylation *in vitro*

**Fig. 2:**
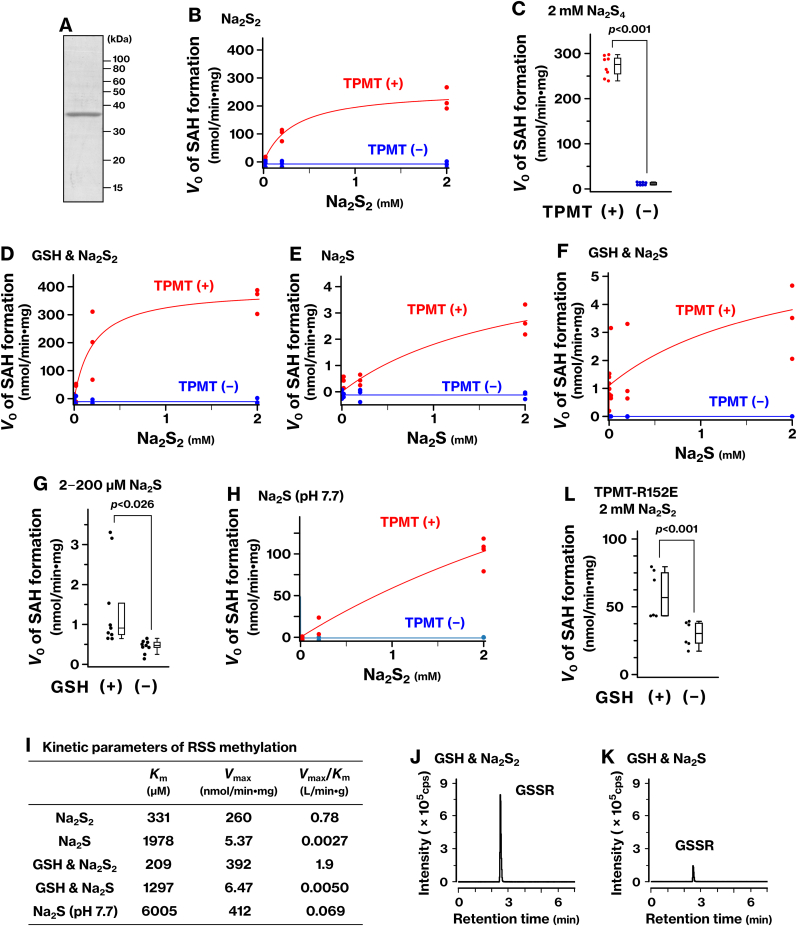
TPMT-mediated methylation of Na_2_S_2_ in the presence of GSH. **(A)** Human TPMT protein was bacterially expressed and purified to near homogeneity. CBB staining is shown. **(B–I)** Na_2_S_2_ (B, D), Na_2_S_4_ (C), or Na_2_S (E–H) was incubated with SAM in the presence (red) or absence (blue) of the recombinant TPMT protein. GSH was included in panels D and F. The reaction was monitored using the MTase-Glo assay. Michaelis–Menten kinetics were analyzed by non-linear least squares fit, and *K*_m_ and *V*_max_ values were calculated (I). Graphs represent results from at least two independent experiments. Statistical significance was assessed using Student's or Welch's *t*-test (C, G). All assays were performed at pH 6.8, except for panel H, which was performed at pH 7.7. **(J, K)** GSH was incubated with Na_2_S_2_ (J) or Na_2_S (K) and derivatized using *N*-iodoacetyltyramine. Shown are the elution profiles at *m/z* 517.14, corresponding to the derivatized GSSH. **(L)** The TPMT-R152E mutant was incubated with Na_2_S_2_ in the presence or absence of GSH, and the methylation reaction was examined. *p*-values were calculated using Student's or Welch's *t*-test.

We assessed whether TPMT catalyzes the methylation of polysulfides using recombinant human TPMT purified from bacteria (Fig. 2A). The methylation reaction of sodium disulfide (Na_2_S_2_) produced *S*-adenosylhomocysteine (SAH) efficiently in the presence, but not in the absence, of TPMT (Fig. 2B and S9C). Kinetic analysis based on the Michaelis–Menten equation yielded a Michaelis constant (*K*_m_) of 331 μM and a maximal reaction velocity (*V*_max_) of 260 nmol/min·mg, corresponding to a catalytic efficiency (*V*_max_/*K*_m_) of 0.78 L/min·g (Fig. 2I). A similar reaction with sodium tetrasulfide (Na_2_S_4_) also produced SAH (Fig. 2C). These results identify disulfide and tetrasulfide species, likely HSS^−^ and HS_4_^−^, as previously unrecognized substrates of TPMT.

### 2.4 TPMT efficiently methylates glutathione persulfide *in vitro*

We examined the methylation of glutathione persulfide (GSS^−^) by TPMT. In these experiments, Na_2_S_2_ was pre-incubated with an excess of glutathione (GSH), allowing the formation of GSS^−^ via a non-redox substitution reaction prior to the addition of TPMT and SAM. The addition of TPMT to the resulting mixture produced SAH (Fig. 2D and S9A). Kinetic parameters under these conditions were *K*_m_ = 209 μM, *V*_max_ = 392 nmol/min·mg, and *V*_max_/*K*_m_ = 1.9 L/min·g (Fig. 2I), indicating higher catalytic efficiency than that observed for Na_2_S_2_ alone. To confirm the formation of GSS^−^, we performed ESI-MS analysis, which detected its *N*-acetyltyramine conjugate (GSSR; Fig. 2J). The amount of GSSR showed a linear relationship with the input concentration of Na_2_S_2_ (Fig. S1), supporting the quantitative conversion of Na_2_S_2_ into GSS^−^. Since the trapping reagent, *N*-iodoacetyltyramine, was added at a concentration comparable to the maximum Na_2_S_2_ concentration tested (up to 2 mM), GSS^−^ formed from Na_2_S_2_ is efficiently derivatized under these conditions. These findings identify GSS^−^ as a novel substrate for TPMT methylation.

### 2.5 GSH promotes TPMT-mediated methylation of H_2_S at low substrate concentrations

We investigated the effects of GSH on TPMT-mediated methylation of H_2_S. In the absence of GSH, TPMT catalyzed the formation of SAH from Na_2_S with low efficiency (Fig. 2E–I and S9D; *K*_m_ = 1978 μM, *V*_max_ = 5.37 nmol/min·mg, *V*_max_/*K*_m_ = 0.0027 L/min·g). Upon addition of GSH, SAH production was enhanced, particularly at low Na_2_S concentrations (2–200 μM) (Fig. 2F and G), although the reaction was less efficient than using Na_2_S_2_. We performed statistical modeling based on enzyme kinetics to evaluate the kinetic parameters and the relative contributions of GSS^−^ and H_2_S as substrates under these conditions (Fig. 2I). The details of this analysis are presented in Supplementary Result 1 (Fig. S1B and C). ESI-MS analysis confirmed the formation of GSS^−^, detected as its *N*-acetyltyramine conjugate GSSR, following incubation of Na_2_S with GSH (Fig. 2K). The yield of GSSR was markedly lower than that produced from Na_2_S_2_ (Fig. 2J and K), suggesting less efficient generation of GSS^−^ from Na_2_S. These results indicate that GSH enhances the TPMT-mediated methylation of H_2_S by facilitating the *in situ* formation of GSS^−^, particularly at low substrate concentrations.

### 2.6 H_2_S methylation by TPMT is enhanced at higher pH, likely due to HS^−^ formation

Given the p*K*_a_ of H_2_S (∼6.9), we investigated whether deprotonation to HS^−^ contributes to its methylation by TPMT. At pH 7.7, where H_2_S is expected to exist predominantly as HS^−^, SAH production was markedly increased compared to that at pH 6.8 (Fig. 2H and I; *K*_m_ = 6005 μM, *V*_max_ = 412 nmol/min·mg, *V*_max_/*K*_m_ = 0.069 L/min·g). The enhanced activity at higher pH is explained by the acid–base equilibrium between H_2_S and HS^−^, suggesting that HS^−^ is the actual substrate recognized by TPMT.

### 2.7 A positively charged active site residue is essential for RSS methylation by TPMT

We tested the methylation activity of a TPMT-R152E mutant, in which a key arginine residue within the active site was substituted with glutamate [11,17,18], to examine the role of electrostatic interactions in substrate recognition. This mutation substantially reduced methylation activity toward both GSS^–^ and HSS^−^ (Fig. 2L). For GSS^–^ methylation, *V*_0_ at 2 mM Na_2_S_2_ decreased from 354 ± 46 to 59 ± 18 nmol/min⋅mg protein (Fig. 2D–L). For HSS^−^, *V*_0_ decreased from 223 ± 39 to 29 ± 9 nmol/min⋅mg (Fig. 2B–L). These findings highlight the importance of a positively charged electrostatic environment within the active site for the recognition and methylation of RSS by TPMT.

### 2.8 Mass spectrometry confirms TPMT-dependent methylation of GSS^−^

**Fig. 3:**
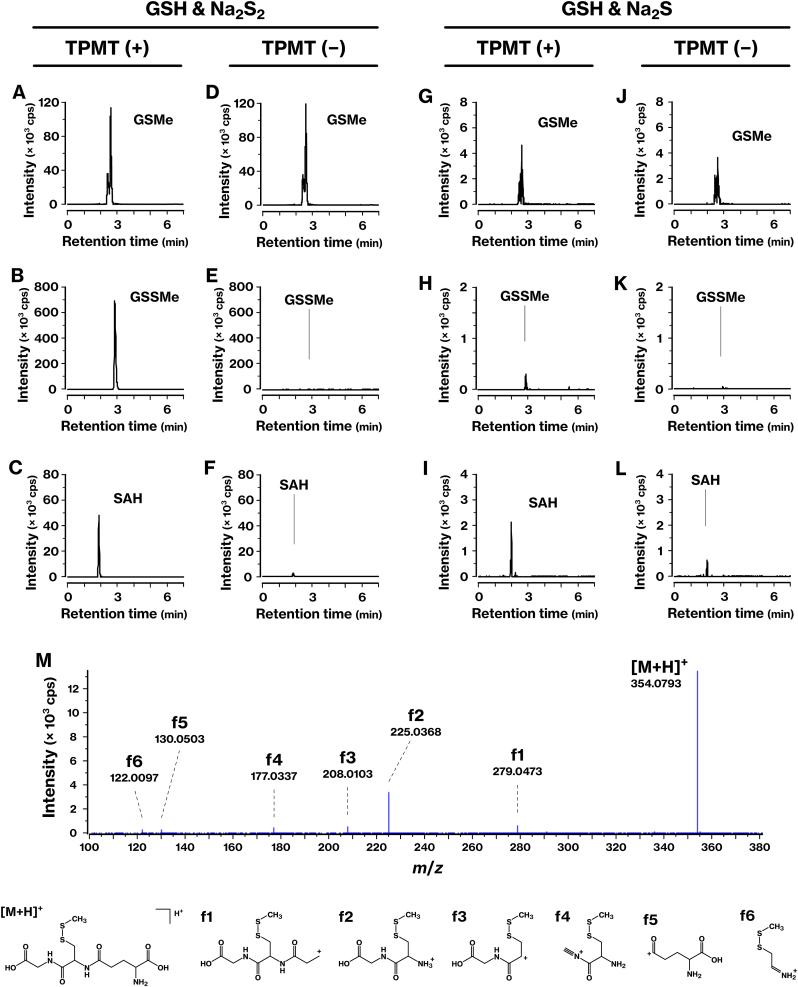
ESI-MS analysis identified GSSMe in TPMT-mediated methylation reactions of Na_2_S_2_ and Na_2_S in the presence of GSH. **(A**–**F)** Na_2_S_2_ was incubated with SAM and GSH in the presence (A–C) or absence (D–F) of TPMT. The reaction was analyzed by LC-Q/TOF-MS. Shown are the extracted ion chromatograms at *m/z* 322.11 (A, D), *m/z* 354.08 (B, E), and *m/z* 385.13 (C, F), corresponding to GSMe, GSSMe, and SAH, respectively. **(G**–**L)** Identical experiments were performed as in A–F, except that Na_2_S was used instead of Na_2_S_2_. **(M)** Na_2_S_2_ was incubated with SAM and GSH in the presence of TPMT, and the reaction was analyzed by LC-Q/TOF-MS to acquire the MS/MS spectrum in the positive ion mode. The precursor and product ion assignments for GSSMe are summarized in Table S1.

We analyzed the TPMT-dependent methylation reaction of GSS^−^ using ESI-MS. In reactions containing TPMT, SAM, and Na_2_S_2_, both methylated glutathione persulfide (GSSMe) and SAH were clearly detected (Fig. 3B, C, E, F). These products were also observed in reactions using Na_2_S as the sulfur source, although signal intensities were 10- to 100-fold lower (Fig. 3H, I, K, L). MS/MS analysis of GSSMe revealed fragment ions with measured *m*/*z* values that closely matched theoretical predictions. Among these, fragments f1–f4 and f6 retained the methylated disulfide moieties (Fig. 3M–Table S1). Methylated glutathione (GSMe) was detected at similar signal intensities in both the presence and absence of TPMT (Fig. 3A–D, G, J), indicating that GSH is not a substrate for TPMT methylation. These results provide direct evidence that TPMT catalyzes the methylation of GSS^−^ *in vitro*.

### 2.9 Binding conformations of HSS^−^ within the TPMT active site identified by simulated annealing–molecular dynamics simulation and fragment molecular orbital method

**Fig. 4:**
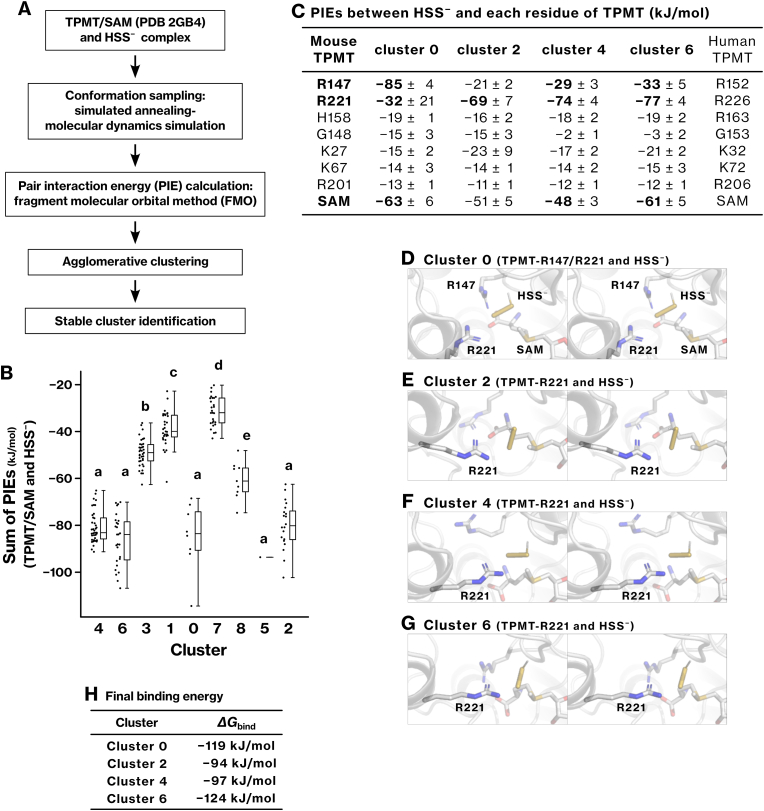
**Identification of TPMT residues involved in HSS^−^ recognition using simulated annealing**–**molecular dynamics (SA-MD) simulation and fragment molecular orbital (FMO) method.** **(A)** HSS^−^ was placed within the active site of mouse TPMT, whose crystal structure was complexed with SAM (PDB 2GB4). Possible binding conformations were explored through SA-MD simulations (Fig. S2A). Sampled conformations were analyzed using the FMO method to calculate pair interaction energies (PIEs) (Fig. S2B and C). These conformations were then grouped through agglomerative clustering based on their PIEs (Fig. S2D and E), allowing for the identification of clusters representing stable binding conformations. **(B)** The sum of PIEs for each conformation was visualized as dot and box-whisker plots for each cluster. Clusters labeled with different letters represent statistically distinct groups as determined by the Tukey–Kramer test (Fig. S2F). **(C)** PIEs were also summed for each TPMT residue and for SAM to identify residues that contribute significantly to the interaction with HSS^−^. The corresponding residues in human TPMT are also shown. SAM refers here to FMO containing the methyl group and S^+^ of SAM (Fig. S2C). **(D**–**G)** Representative binding conformations of HSS^−^ from clusters 0, 2, 4, and 6 are illustrated. **(H)** The most favorable conformation from each cluster was further optimized using the FMO2-DFTB3/PCM method. The final binding free energy (*ΔG*_bind_) between HSS^−^ and the TPMT/SAM complex was defined as *ΔG*_bind_ = *E*_TPMT/SAM/HSS_ – (*E*_TPMT/SAM_ + *E*_HSS_) and calculated using the FMO3-DFTB3/PCM method.

To investigate the molecular basis of HSS^−^ recognition by TPMT, we performed simulated annealing–molecular dynamics (SA-MD) simulations and fragment molecular orbital (FMO) calculations (Fig. 4A). The SA-MD simulation identified multiple stable binding conformations of HSS^−^ within the active site of the TPMT complexed with SAM (Fig. S2A), which were grouped into clusters on the basis of pair interaction energies (PIEs) between HSS^−^ and the TPMT/SAM complex (Fig. 4B; S2B–E). Details of conformation sampling and analysis are provided in the *Identification of stable binding conformations* section in Methods. Clusters 0, 2, 4, and 6 exhibited the largest binding energies that were statistically distinct from those of other clusters (Fig. 4B–S2F).

FMO analysis revealed an interaction between HSS^−^ and the SAM fragment (Fig. 4C), which is defined to include the sulfonium cation and the methyl group in the FMO method (Fig. S2C). In cluster 0, HSS^−^ was stabilized by interactions with two arginine residues, R147 and R221, corresponding to human TPMT R152 and R226 (Fig. 4C and D). In clusters 2, 4, and 6, R221 provided the dominant interaction (Fig. 4C–E–G). Electrostatic interactions accounted for the majority of the interaction energy (Table S2). These results suggest that HSS^−^ is recognized and stabilized within the active site through coordinated interactions with arginine residues and SAM, consistent with the reduced methylation activity observed *in vitro* for the TPMT-R152E mutant (Fig. 2L).

The best binding conformation of HSS^−^ was further optimized using the FMO2-DFTB3/PCM method, and binding energy (*ΔG*_bind_) was calculated using the FMO3-DFTB3/PCM method. The resulting *ΔG*_bind_ values ranged from −94 to −124 kJ/mol (Fig. 4H), supporting the thermodynamic feasibility of HSS^−^ binding within the TPMT active site.

### 2.10 Energetic and structural insights into GSS^−^ recognition by TPMT from SA-MD simulations and FMO analysis

**Fig. 5:**
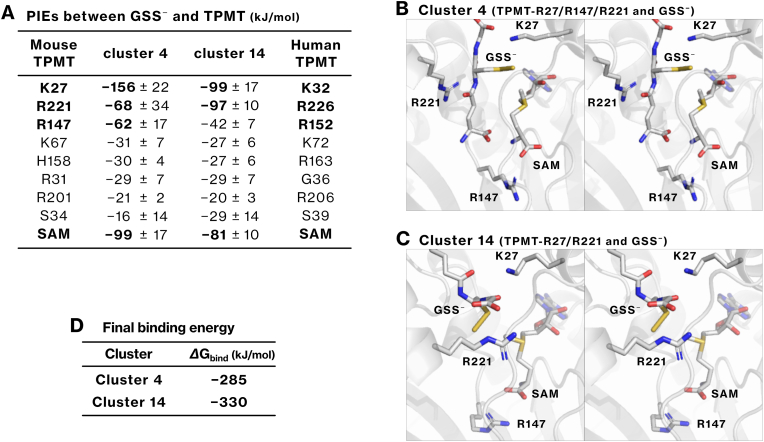
Possible binding conformations of GSS^−^ within the active site of TPMT were identified using SA-MD simulation and the FMO method. **(A)** Using the same procedure as described in Fig. 4A, stable binding conformations of GSS^−^ within the active site of the TPMT/SAM complex were identified (Figs. S3 and S4). The PIEs of each TPMT residue and SAM are shown to evaluate their contributions to the interaction with GSS^−^. The corresponding residues in human TPMT are also indicated. **(B, C)** The binding conformations of GSS^−^ in clusters 4 and 14 are depicted. **(D)** The best conformations in clusters 4 and 14 were geometry-optimized, and the final binding energy, *ΔG*_bind_, of GSS^−^ and the TPMT/SAM complex was calculated using the FMO3-DFTB3/PCM method.

We investigated the binding conformations of GSS^−^ within the active site of TPMT through SA-MD simulations and FMO analysis, as described in the Methods section. From these analyses, clusters 4 and 14 were identified as stable conformational clusters (Figures S3A–C, S4A–C). In both clusters, GSS^−^ was stabilized by coordinated interactions with K27, R147, R221, and SAM (Fig. 5A–C). R147 interacted with SAM (Fig. 5B and C), consistent with previous crystallographic data [17]. The relative contribution of R147 to GSS^−^ binding was lower than that observed for HSS^−^ (Fig. 5A–C), agreeing with the *in vitro* observation that the TPMT-R152E mutant retained higher methylation activity toward GSS^−^ than HSS^−^ (Fig. 2L). These results indicate that while TPMT employs arginine residues to recognize both HSS^−^ and GSS^−^, the mode of interaction differs: HSS^−^ binds via the negatively charged sulfur atom, whereas GSS^−^ engages through its GSH moiety.

We further evaluated the strength and stability of GSS^−^ binding. The binding energies, *Δ*G_bind,_ for clusters 4 and 14 were calculated to be −285 and −330 kJ/mol, respectively (Fig. 5D). In conventional MD simulations without distance restraints, GSS^−^ remained stably bound within the active site and could reassociate with SAM even after transient dissociation (Fig. S5A−D). In contrast, HSS^−^ dissociated readily from the active site (Fig. S7). These results indicate that GSH enhances stable substrate recognition by TPMT and that GSS^–^ binds more stably than HSS^−^, in line with *in vitro* results indicating more efficient methylation of GSS^−^ than HSS^−^ (Fig. 2I).

### 2.11 TPMT efficiently methylates selenite-derived anions but not MeSe^−^*in vitro*

**Fig. 6:**
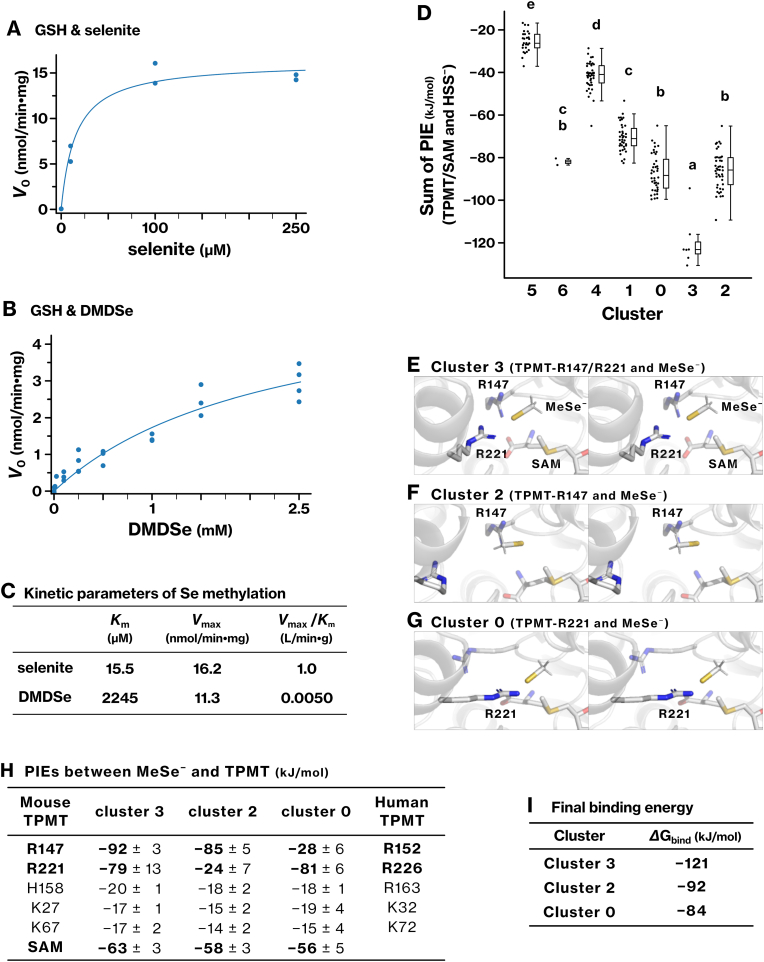
Quantitative analysis of Se methylation reaction catalyzed by TPMT and possible binding conformations of MeSe^−^. **(A**–**C)** Sodium selenite (A) or dimethyldiselenide (DMDSe) (B) was incubated with SAM, GSH, and TPMT protein. The reaction was monitored using the MTase-Glo assay. Michaelis–Menten kinetics were analyzed by non-linear least squares fit, and *K*_m_ and *V*_max_ values were determined (C). The graphs represent the results from more than two independent experiments. **(D)** Using the same procedure as described in Fig. 4A, binding conformations of methylselenide anion (MeSe^−^) within the active site of the TPMT/SAM complex were sampled and classified into seven clusters (Fig. S6). The sum of PIEs of each conformation was plotted for each cluster. Clusters labeled with different letters represent statistically distinct groups, as determined by the Tukey–Kramer test. **(E**–**G)** Representative binding conformations of MeSe^−^ in clusters 3, 2, and 0 are shown. **(H)** PIEs were summed for each TPMT residue to identify residues that significantly contributed to the interaction with MeSe^−^. The corresponding residues in human TPMT are also indicated. **(I)** The best conformations in clusters 3, 2, and 0 were geometry-optimized, and the final binding energy, *ΔG*_bind_, of MeSe^−^ and the TPMT/SAM complex was calculated using the FMO3-DFTB3/PCM method.

To contextualize TPMT activity toward RSS, we compared it with its known activity toward Se-containing compounds. In the presence of GSH, selenite is reduced to glutathione perselenide (GSSe^−^) and hydroselenide (HSe^−^) [4], either or both of which serve as substrates for TPMT, as evidenced by SAH production (Fig. 6A–S9B and S9E). The estimated kinetic parameters were *K*_m_ = 15.5 μM, *V*_max_ = 16.2 nmol/min·mg, and *V*_max_/*K*_m_ = 1.0 L/min·g (Fig. 6C). By contrast, dimethyldiselenide (DMDSe) was methylated less efficiently. Although the GSH-mediated reduction of DMDSe generated methylselenide anion (MeSe^−^), the reaction catalyzed by TPMT showed a much higher *K*_m_ (2245 μM) and a lower catalytic efficiency (*V*_max_/*K*_m_ = 0.0050 L/min·g) (Fig. 6B, C and S9F). These results indicate that TPMT efficiently methylates selenite-derived anions, such as GSSe^−^ or HSe^−^, whereas MeSe^−^ is a poor substrate.

### 2.12 TPMT accommodates MeSe^−^ in binding conformations similar to HSS^−^

To understand the mechanistic basis for the different methylation efficiencies for Se-containing compounds, we investigated the binding conformations of MeSe^−^ within the active site of TPMT using SA-MD simulations and FMO analysis. The sampled conformations were grouped into seven clusters (Fig. S6A), among which cluster 3 exhibited the strongest interaction energy (Fig. 6D; S6A, B). In cluster 3, MeSe^–^ was stabilized through direct interactions with R147 and R221 (Fig. 6E). R147, R221, and SAM made the largest contributions to the binding energy (Fig. 6H). In other clusters, R147 and R221 contributed to different extents, with R147 involved in cluster 2 and R221 in cluster 0 (Fig. 6F−H). These findings are consistent with our previous experimental data showing that the human TPMT-R152E mutant, corresponding to mouse R147E, was completely deficient in catalyzing the methylation of MeSe^–^ derived from DMDSe [11]. MeSe^–^ showed the largest binding energy in cluster 3 (*ΔG*_bind_ = −121 kJ/mol) (Fig. 6I), which is comparable to that of HSS^−^ (Fig. 4H). This suggests that differences in catalytic efficiency may not be explainable by binding strength alone.

### 2.13 HOMO energy levels are comparable among RSS and selenide anions

**Fig. 7:**
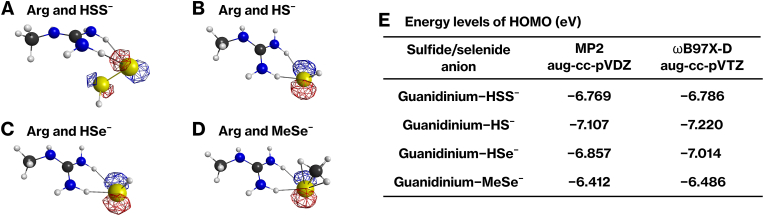
Energy levels of the highest occupied molecular orbitals (HOMOs) of HS^−^, HSS^−^, HSe^−^, and MeSe^−^ in complexes with a guanidinium group. **(A−D)** HS^−^, HSS^−^, HSe^−^, and MeSe^−^ were each complexed with methylated guanidinium, and their geometries were optimized using the MP2/aug-cc-pVDZ level of theory. The highest occupied molecular orbitals (HOMOs) corresponding to each molecule are depicted. **(E)** HOMO energy levels were calculated using the delta self-consistent field method at the MP2/aug-cc-pVDZ and ωB97X-D/aug-cc-pVTZ levels of theory.

To assess the intrinsic nucleophilic reactivity of TPMT substrates, we calculated the highest occupied molecular orbital (HOMO) energy levels for HSS^−^, HS^−^, HSe^−^, and MeSe^−^ using quantum chemical methods. Each anion was complexed with a guanidinium ion, representing the electrostatic environment of an arginine side chain within the TPMT active site, followed by geometry optimization. In all four complexes, HOMO was primarily located on the sulfur or Se anion (Fig. 7A–D), indicating that the nucleophilic center is retained upon complex formation. The calculated HOMO energy levels were similar across all species (Fig. 7E), suggesting comparable intrinsic reactivity. These results suggest that the differences in methylation efficiency are not solely attributable to electronic factors.

### 2.14 Differential susceptibility to steric hindrance of MeSe^−^ and HSS^−^ upon nucleophilic attack

**Fig. 8:**
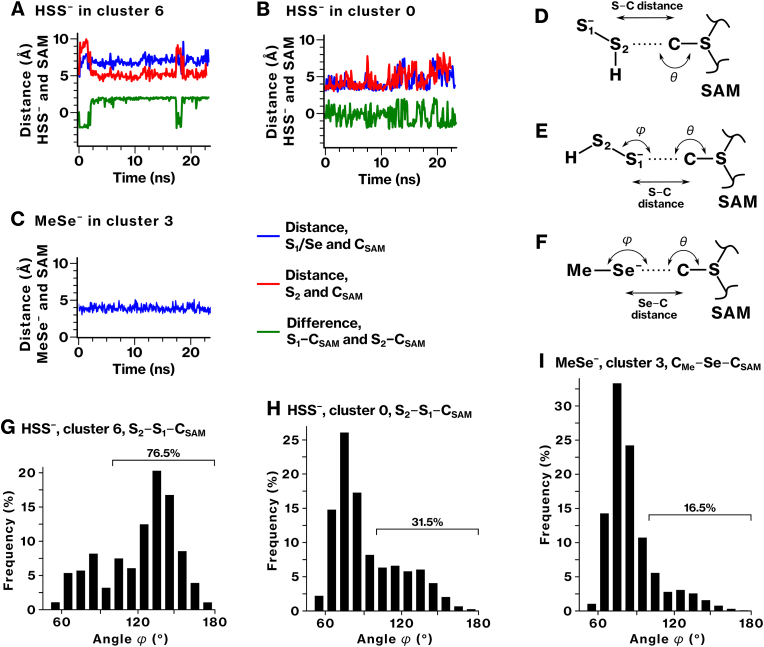
Dynamics of HSS^−^ and MeSe^−^ in the active site of TPMT. **(A**–**C)** Positional restraints were removed, and the dynamics of HSS^−^ and MeSe^−^ within the active site of the TPMT/SAM complex were investigated using conventional MD simulation. The distances between the carbon atom in the methyl group of SAM (C_SAM_) and either the sulfur atoms in HSS^−^ (S_1_ and S_2_) or the Se atom are shown in blue and red. The difference between S_1_−C_SAM_ and S_2_−C_SAM_ is shown in green (A, B). Representative results from ten independent MD simulations are presented; the remaining data are provided in Figs. S7 and S8. **(D**–**I)** The dynamics of HSS^−^ and MeSe^−^ were analyzed over the initial 20 ns of the production MD simulations. S/Se–C distance and angles *θ* and *φ* were defined as depicted (D–F). Frames with the S/Se–C distance within 4 Å and the *θ* angle ranging from 150° to 180° were extracted for analysis of angle *φ*. In these frames, angle *φ* is shown in the histograms (G–I). Frames where *φ* was within the range of 100 to 180° were counted.

According to frontier molecular orbital theory, nucleophilic reactivity is influenced not only by electronic properties but also by steric accessibility. As HOMO energy levels were similar among the tested species, we next examined whether steric effects could account for the observed differences in methylation efficiency. We performed MD simulations of the binding conformations of ligands HSS^−^ and MeSe^−^ within the TPMT active site. Three representative structures, two for HSS^−^ and one for MeSe^−^, were selected on the basis of SA-MD clustering results (Fig. 4D, G, 6E). Conventional MD simulations without distance restraints revealed a dynamic association of ligands with the TPMT active site (Fig. 8A−C, S7, S8; see Supplementary Result 2 for details). These results indicate the conformational behavior of ligands within the active site prior to transition state formation, providing a structural basis for evaluating whether these conformations are geometrically suitable for nucleophilic attack.

To evaluate steric constraints on nucleophilic attack, we analyzed the spatial arrangement of the nucleophilic center (S^−^ or Se^−^) relative to the methyl group of SAM using two geometric parameters: *θ*, reflecting the alignment for nucleophilic attack, and *φ*, indicative of steric hindrance from surrounding groups (Fig. 8D−F). We extracted MD frames that satisfied the geometric criteria, namely, an S/Se–C_SAM_ distance of less than 4 Å and an angle *θ* within 150° to 180°, as spatial arrangements conducive to nucleophilic attack (Table S3; see Supplementary Result 2 for details). Among these, the histogram of angle *φ* (S_2_–S_1_–C_SAM_) for HSS^−^ in cluster 6 peaked in the 120° to 150° range, and 76.5% of the extracted frames fell within the optimal *φ* range (100°–180°), indicating minimal steric hindrance and favorable geometry for methyl transfer (Fig. 8G and H; Table S3). In contrast, the *φ* distribution of MeSe^−^ was centered around 70° to 80°. Only 16.5% of the extracted frames fell within the optimal *φ* range (100°–180°), suggesting significant steric hindrance from the adjacent methyl group (Fig. 8I; Table S3). These results indicate that steric accessibility, rather than electronic properties, plays a key role in determining methylation efficiency, and that nucleophilic attack by MeSe^−^ is highly susceptible to steric hindrance imposed by its neighboring methyl group. This is consistent with the kinetic data showing a significantly low *V*_max_/*K*_m_ value for MeSe**^−^** compared to HSS**^−^** (Fig. 6C for MeSe**^−^**; Fig. 2I for HSS**^−^**).

## 3 Discussion

RSS are known to provide cellular protection against oxidative and electrophilic stress [3]. However, accumulating evidence indicates that the excessive levels of RSS are cytotoxic. The cytotoxicity of H_2_S is well documented [19,20]. Persulfides and polysulfides have also been implicated in oxidative stress and cytotoxicity [21,22]. Notably, excess polysulfur-containing cysteine derivatives are actively exported from cells to mitigate RSS cytotoxicity [23,24]. Furthermore, knockout mice lacking genes involved in the oxidative clearance of RSS exhibit phenotypes consistent with cytotoxicity caused by RSS accumulation [25,26]. These findings establish that excessive levels of RSS can exert toxic effects on biological systems.

We demonstrated that TPMT catalyzed the methylation of polysulfides, persulfides, and HS^−^ (Fig. 10B). This SAM-dependent methylation establishes a novel, non-oxidative pathway for RSS detoxification. *In vitro* biochemical assays, mass spectrometry, cellular experiments, and a mouse model collectively demonstrated that TPMT reduced intracellular RSS levels and mitigated RSS-induced cytotoxicity. To our knowledge, this is the first report identifying a methyltransferase responsible for the methylation of RSS. Our findings indicate that mammals possess an active, energy-consuming metabolic mechanism for RSS elimination, which complements existing energy-independent oxidative pathways, thereby expanding the known landscape of redox regulation.

Our findings not only broaden the biological significance of TPMT but also raise the possibility that TPMT polymorphisms may influence cellular redox balance through altered RSS metabolism. Notably, H_2_S and related sulfur species are increasingly recognized as pharmacologically active compounds and are being explored in clinical contexts [27,28]. In this context, variation in TPMT activity may have implications for how cells respond to exogenous RSS-modulating agents. Further studies will be required to determine whether TPMT polymorphisms influence the biological or pharmacological actions of therapeutic RSS donors.

Our findings suggest that RSS represent endogenous substrates of TPMT, even though TPMT has long been recognized for its role in the S-methylation of thiopurine drugs [29,30]. Se species, derived from the essential trace element, have been identified as endogenous substrates of TPMT [[10], [11], [12], [13]]. TPMT methylates RSS *in vitro* with efficiency comparable to that for Se species (Figs. 2I and 6C), and its inhibition increases cellular susceptibility to RSS-induced cytotoxicity (Fig. 1D). Consistently, urinary excretion of TMS was markedly decreased in TPMT^−/−^ mice (Fig. 1A). These results suggest that RSS constitute another endogenous substrate and that TPMT detoxifies excess RSS through methylation *in vivo*.

In previous works, it remained unclear whether urinary TMS represents methylation products of RSS, whose levels may reflect endogenous H_2_S metabolism, or whether it is merely another sulfur metabolite [[14], [15], [16]]. The identification of TPMT as RSS methyltransferase, together with the phenotype of KO mice, revealed that RSS is converted to TMS and subsequently excreted in urine.

TPMT-mediated methylation is essential as a broadly acting detoxification pathway that operates across diverse substrates, including Se species, RSS, and thiopurine drugs. Our data demonstrate that TPMT is critical in mitigating the cytotoxic effects of excess RSS *in vivo* (Fig. 1). In Se metabolism, excess Se, but not the nutritional level, is directed to the TPMT-initiated sequential methylation [10,11,31]. In patients receiving 6-MP, TPMT polymorphisms, relatively common in the population, can result in severe adverse effects, such as myelosuppression [29,30]. Therefore, TPMT-mediated pathways are crucial when cytotoxic substrates reach excessive levels.

TPMT-mediated methylation of Se and RSS shares similarities and differences. Like Se, RSS undergoes consecutive methylation that facilitates its excretion. As we have previously demonstrated, Se is methylated by TPMT and INMT to form TMSe, one of the major urinary Se metabolites [10,31,32]. Similarly, TPMT-mediated methylation of RSS produces methanethiol (MeSH), which is further methylated by INMT and/or METTL7B to generate TMS (Fig. 10C) [[33], [34], [35]]. TMS is excreted in urine and detected at several hundred nanomolar concentrations [[14], [15], [16]]. Although TMS was not directly detected in the present enzymatic assays, the previous studies demonstrated that INMT catalyzed the methylation of dimethyl sulfide [35]. Furthermore, the urinary levels of TMS were influenced by single nucleotide polymorphisms of *INMT* [36], supporting a model in which sequential methylation by TPMT and INMT led to the formation of TMS. These findings indicate that TPMT and INMT play parallel roles in the detoxification and excretion of both Se and RSS. However, a key distinction lies in their end products: TMSe is chemically stable and biologically inert [[37], [38], [39]], whereas TMS is highly reactive and commonly used as a methylating agent in synthetic chemistry [40]. Thus, although both pathways involve methylation, the metabolites diverge significantly in terms of chemical stability and biological implications.

Oxidation- and methylation-based pathways cooperate to regulate intracellular RSS levels but fulfill distinct physiological roles. In Se metabolism, physiological levels of Se are converted into selenosugars, whereas excess Se is excreted as TMSe [31]. By analogy, oxidation likely represents the homeostatic route for basal RSS turnover, whereas methylation serves as a detoxification pathway for eliminating excess RSS. This interpretation is supported by knockout phenotypes. Individuals carrying TPMT loss-of-function alleles are generally asymptomatic under physiological conditions, despite markedly reduced or absent TPMT activity [29,30]. In our analysis, TPMT^−/−^ mice appeared to grow normally. In contrast, loss of SQOR or ETHE1 results in severe mitochondrial dysfunction and early lethality due to impaired oxidative sulfur metabolism [25,26]. These contrasting phenotypes underscore the housekeeping role of the oxidative system in maintaining RSS homeostasis.

HS^−^ and GSS^−^ serve as branching points between TPMT-mediated inactivation and oxidation-mediated excretion of RSS. Sulfur oxidation is driven by several enzymes, including SQOR, ETHE1, and sulfite oxidase [5,41]. SQOR facilitates the oxidative elimination of HS^−^ by conjugating it with GSH to form GSS^−^, which is subsequently oxidized by ETHE1 (Fig. 10A). Because HS^−^ and GSS^−^ are also TPMT substrates, these molecules represent key nodes that determine whether sulfur is methylated or oxidized. Oxidation by ETHE1 and sulfite oxidase results in sulfate excretion in urine. In contrast, TPMT-mediated methylation inactivates persulfide while retaining sulfur in the form of methylated polysulfide, which can be reactivated to persulfide through reversible exchange with HS^−^. Thus, TPMT-dependent methylation modulates RSS bioactivity without reducing the overall sulfur pool, providing an energy-dependent alternative to irreversible oxidative clearance.

The functional complementarity between oxidation and methylation is also facilitated by the subcellular localization of these enzymes: SQOR and ETHE1 reside in mitochondria [5,41], whereas TPMT is a cytoplasmic enzyme [30]. Excess H_2_S inhibits cytochrome *c* oxidase (complex IV) in mitochondria [19,20]. Under conditions like hypoxia or sulfide overload, coenzyme Q is markedly reduced [42], and the deficiency in coenzyme Q limits SQOR-mediated H_2_S oxidation [43,44]. Therefore, RSS is oxidized in mitochondria under physiological conditions; however, excess RSS induce mitochondrial dysfunction. The spatial separation provides a safeguard mechanism, enabling TPMT to mitigate toxic RSS accumulation even when mitochondrial function is impaired.

H_2_S is likely methylated by TPMT in the form of HS^−^ *in vivo*, as suggested by the *V*_max_/*K*_m_ values at pH 6.8 and 7.7 (Fig. 2E–H). Given that cytosolic and mitochondrial pH ranges from 7.1 to 8.0 [45,46], H_2_S predominantly exists as HS^−^ under physiological conditions. Together, these findings suggest that TPMT is a primary methyltransferase responsible for inactivating RSS, including HS^−^. This point is further discussed in Supplementary Discussion 1, under the section *TPMT is the primary methyltransferase for H*_*2*_*S metabolism in vivo*.

Our data indicate that TPMT mediates the methylation of GSS^−^. Kinetics analysis showed that GSS^−^ was a more favorable substrate for TPMT-mediated methylation than HSS^−^ (Fig. 2I). Consistently, *in silico* modeling revealed that the binding energy of GSS^−^ was approximately three times higher than that of HSS^−^ (Figs. 4H and 5F). Moreover, GSH enhanced the methylation reaction involving Na_2_S (Fig. 2E–G), and the kinetic profiles were well explained by the formation and methylation of GSS^–^ (Fig. 2F–S1B; Supplementary Result 1). ESI-MS analysis also indicated the formation and subsequent methylation of GSS^–^ (Fig. 2J and K; 3B, H). Collectively, these findings support the direct methylation of GSS^−^, as well as HS^−^ and HSS^−^, by TPMT. These findings also suggest a role for GSH in the methylation of Se compounds. This point is further discussed in Supplementary Discussions 2, under the section *GSH enhances substrate recognition by TPMT in the methylation of Se compounds*.

TPMT recognizes negatively charged substrates through interactions with basic residues within the active site during RSS methylation, as proposed for Se compounds in our previous study [11]. Our *in silico* analysis predicted the binding conformations of HSS^−^ within the active site of TPMT (Fig. 4). Specifically, HSS^−^ was recognized by arginine residues within the active site, including mouse TPMT-R147 and R221, which correspond to R152 and R226 in human TPMT (Fig. 4D–G). Comparable binding conformations were also observed for MeSe^–^ (Fig. 6E−G). Consistently, human TPMT-R152 mutants exhibited reduced methylation activity toward the non-methylated and monomethylated forms of Se in our earlier study [11]. The TPMT-R152E mutation also reduced methylation activity toward GSS^−^ and HSS^−^ (Fig. 2L). Similarly, a previous biochemical study disclosed that mouse TPMT-R147 and R221 were involved in the recognition of 6-MP [17]. These findings suggest that TPMT employs a similar recognition mechanism for HSS^−^, Se compounds, and 6-MP.

Differences in methylation efficiency among HSS^−^, HS^−^, and MeSe^−^ cannot be attributed solely to their binding energies or HOMO energy levels. Quantum chemical calculations of HOMO energies revealed comparable intrinsic nucleophilic reactivity of HSS^−^, HS^−^, HSe^−^, and MeSe^−^ (Fig. 7E). Furthermore, HSS^−^ and MeSe^−^ exhibited similar binding energies (Figs. 4H and 6I), and HS^−^ and HSe^−^ were also expected to exhibit comparable values, as these interactions were primarily driven by electrostatic interactions involving arginine residues and SAM (Table S2). In contrast, the catalytic efficiencies, *V*_max_/*K*_m_, for HSS^−^, HS^−^, and MeSe^−^ differed by approximately one order of magnitude, with HSS^−^ exhibiting the highest value (Figs. 2I and 6C). These findings indicate that factors beyond binding affinity and electronic structure critically influence methylation efficiency.

**Fig. 9:**
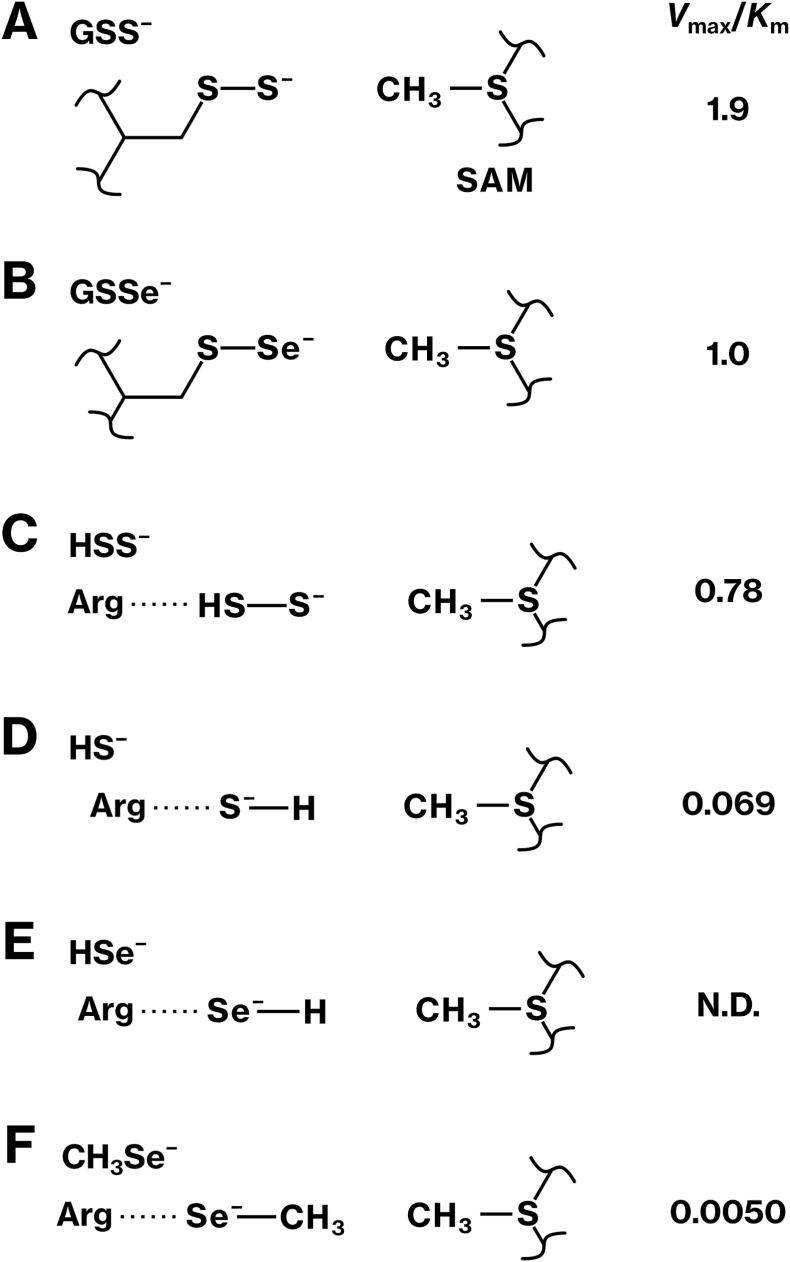
Possible steric hindrance and methylation efficiency. **(A**–**F)** Possible binding conformations of ligands within the TPMT active site are listed. The catalytic efficiency, *V*_max_/*K*_m_ (L/min·g), is also indicated. Arg, arginine residue. N.D., not determined.

Steric hindrance around sulfide and selenide anions appears to be a major determinant of methylation efficiency for HSS^−^, HS^−^, and MeSe^−^. MD simulations of MeSe^–^ suggested that the methyl group of MeSe^−^ hinders the nucleophilic attack by Se^−^ on SAM (Figs. 8I and 9F; Table S3). In contrast, HSS^−^ adopts conformations more favorable for nucleophilic attack without significant steric hindrance, with the SH moiety interacting with arginine, leaving the S^−^ anion accessible (Figs. 8G and 9C; Table S3). This is supported by the observation that both S^−^ and SH groups can interact with arginine residues (Fig. 8A and B), and the HOMO is primarily localized on S^−^ (Fig. 7A). In GSS^−^ and GSSe^−^, steric hindrance is minimal (Fig. 5B, C, 9A, B). HS^−^ and HSe^−^ likely bind to arginine residues in a manner similar to HSS^−^ and MeSe^−^ (Fig. 9D and E). Notably, catalytic efficiency, *V*_max_/*K*_m_, increases in the order of MeSe^−^, HS^−^, HSS^−^, and GSS^−^/GSSe^−^, which inversely correlates with the degree of predicted steric hindrance (Fig. 9A–F). These findings support the conclusion that steric effects near the nucleophilic center are the key determinants of TPMT-mediated methylation efficiency.

**Fig. 10:**
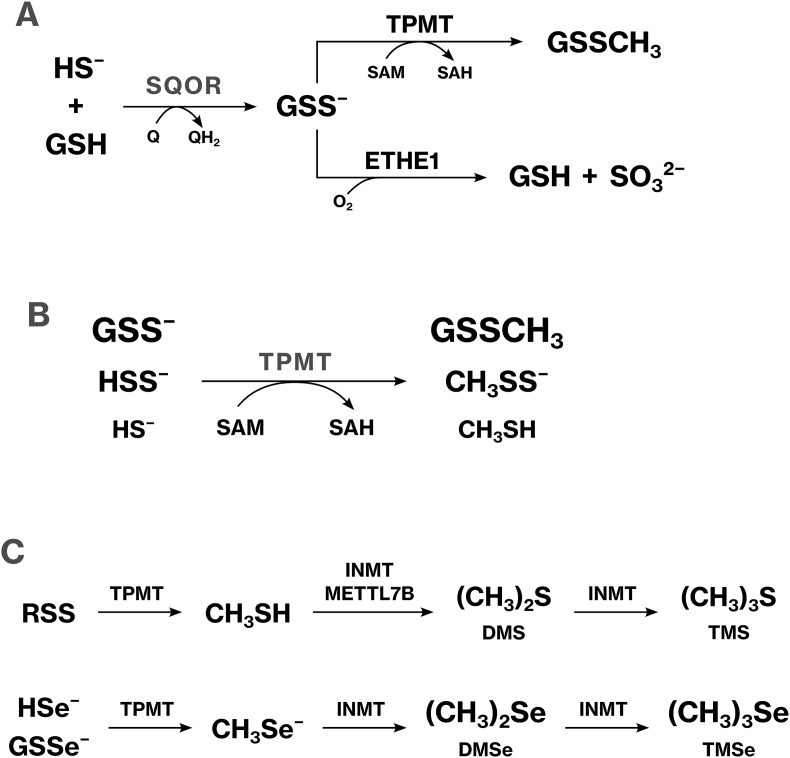
TPMT-mediated methylation of RSS. **(A)** A branch point in the oxidative and methylation pathways of RSS. **(B)** TPMT-mediated methylation of RSS. **(C)** Trimethylation of Se and RSS.

RSS provide cellular protection against oxidative and electrophilic stress, but their excessive accumulation can be toxic. Despite their physiological significance, the regulatory mechanisms that maintain RSS homeostasis remain incompletely understood. Our findings identify TPMT-mediated methylation as a key non-oxidative mechanism for regulating intracellular levels of RSS. By targeting HS^−^, HSS^−^, and GSS^−^, TPMT offers an energy-dependent alternative to mitochondrial oxidative pathways mediated by enzymes, such as SQOR and ETHE1. Physiologically, TPMT-mediated methylation plays a critical role in enhancing cell survival under conditions of excess RSS by facilitating their metabolic detoxification. Moreover, TPMT promotes urinary excretion of RSS as its methylated form, TMS. Collectively, our data establish TPMT-mediated methylation as a key mechanism that protects cells from excessive RSS and complements mitochondrial oxidative pathways. Because TPMT polymorphisms are already established as the biomarker for predicting thiopurine toxicity, variation in TPMT may influence cellular responses to exogenous RSS donors. Future studies should explore how the interplay between methylation and oxidation is modulated *in vivo* and how the dysregulation of TPMT contributes to diseases associated with RSS imbalance.

## 4 Methods

### 4.1 Protein expression and purification

Human TPMT (NCBI NM_000367) was synthesized and cloned into pET29b by Twist Bioscience (USA). A histidine tag was added to the C-terminus of the open reading frame. Expression and purification of recombinant protein were described previously [11,47]. Briefly, the plasmid was transformed into a BL21(DE3) strain. The recombinant bacteria were cultured in an LB medium, and the expression was induced with 0.5 mM IPTG at 37 °C for 3 h. Bacterial cells were collected by centrifugation and lysed in a lysis buffer [20 mM NaPi (pH 6.8), 300 mM NaCl] supplemented with a proteinase inhibitor cocktail. The bacterial lysate was loaded on Talon resin (Takara Bio, Japan, Cat. No. 635501) with a purification buffer [20 mM NaPi (pH 6.8), 300 mM NaCl, 0.1% Triton X-100], and the recombinant protein was eluted with the purification buffer containing 300 mM imidazole. The eluted fraction was dialyzed against a dialysis buffer [50 mM Tris-HCl (pH 7.4), 100 mM NaCl, 0.1% Triton X-100, 50% glycerol]. Protein concentration was quantified by the Bradford assay (BioRad, #5000006).

### 4.2 Quantitative evaluation of methylation reaction *in vitro*

Quantitative evaluation of methylation reaction was performed using the MTase-Glo methyltransferase assay kit (Promega Corporation, USA, Cat. No. V7601) by monitoring the conversion of SAM into *S*-adenosylhomocysteine (SAH) by methyltransferases, as described previously with modifications [11]. The reaction mixture was composed of 20 mM sodium phosphate buffer (pH 6.8), 0.01% Triton X-100, and 5 μg/mL of recombinant methyltransferases. SAM was added to the reaction mixture at a final concentration of 25 μM immediately before the reaction. GSH was included in the reactions at a final concentration of 5 mM where specifically noted. The substrate was either sodium disulfide (Na_2_S_2_), sodium tetrasulfide (Na_2_S_4_), sodium sulfide (Na_2_S), sodium selenite (Na_2_SeO_3_), or dimethyldiselenide (DMDSe). Na_2_S_2_ (Dojindo Laboratories, Japan, Cat. No. SB02), Na_2_S_4_ (Dojindo, Cat. No. SB04), or Na_2_S·5H_2_O (FUJIFILM Wako Pure Chemical Corporation, Japan, Cat. No. 197-13752) was included in the reaction at final concentrations of 0 μM, 20 μM, 200 μM, and 2.0 mM. Sodium selenite (Nacalai Tesque, Cat. No. 3182402) was used as a substrate at final concentrations of 0 μM, 10 μM, 100 μM, and 250 μM. DMDSe (Tokyo Chemical Industry, Cat. No. D3290) was used at final concentrations of 0 μM, 10 μM, 100 μM, 250 μM, 500 μM, 1.5 mM, and 2.5 mM. Glutathione persulfide, sodium disulfide, and sodium sulfide were incubated with GSH at room temperature for 5 min and then added to the reaction mixture. The reaction involving Na_2_S_2_ and Na_2_S_4_ proceeded at 37 °C for 5 min, and those involving selenite and Na_2_S proceeded at 37 °C for 30 min. The reactions were terminated by adding trifluoroacetic acid at a final concentration of 0.1%. The amount of SAH in the reaction mixture was quantified in accordance with the manufacturer's instructions. Chemifluorescence was quantified using Spectramax iD3 (Molecular Devices, USA).

### 4.3 Estimation of kinetic parameters

The kinetic parameters of the methylation reaction were estimated using the Michaelis–Menten equation, as described previously [11]. *K*_m_ and *V*_max_ values were estimated using a non-linear least squares fit. Calculations were performed using a minimize function in the optimize module in SciPy 1.11.4. A fitting curve was generated using Matplotlib 3.9.2 and seaborn 0.13.2.

### 4.4 Linear combination of two Michaelis–Menten equations

The reaction involving Na_2_S and GSH was analyzed using a linear combination of two Michaelis–Menten equations. The Michaelis–Menten equation was defined using the substrate concentration *S* and the initial reaction rate *v*_0_ as follows:

(equation 1) $$v_{0}\left(S\right)=MM\left(S;\;K_{m},V_{\max}\right)=\frac{V_{\max}\cdot S_{0}}{K_{m}+S_{0}}$$

The apparent initial reaction rate, $v_{0}^{app}\text{,}$ was defined using the linear combination of Michaelis–Menten equations for GSS^–^, $v_{0}^{GSS}$, and for H_2_S, $v_{0}^{H2S}$.

(equation 2) $$v_{0}^{app}=v_{0}^{GSS}+v_{0}^{H2S}$$

(equation 3) $$v_{0}^{GSS}\left(S;\;C_{1}\right)=\left\{ \begin{array}{c} MM\left(S;\;K_{m}^{GSS},V_{\max}^{GSS}\right)\;if\;S\;<\;C_{1}\\ MM\left(C_{1};\;K_{m}^{GSS},V_{\max}^{GSS}\right)\;if\;S\geq C_{1} \end{array}\right.$$

(equation 4) $$v_{0}^{H2S}\left(S;\;C_{1}\right)=\left\{ \begin{array}{c} 0\;if\;S\;<\;C_{1}\\ MM\left({S-C}_{1};\;K_{m}^{H2S},V_{\max}^{H2S}\right)\;if\;S\geq C_{1} \end{array}\right.$$

The *K*_m_ and *V*_max_ values for GSS^–^ and H_2_S were calculated beforehand from the reaction involving Na_2_S_2_ and GSH and the reaction involving Na_2_S only. $C_{1}$ is an upper limit of the concentration where H_2_S is oxidatively converted into GSS^−^. The calculation was carried out under the assumption that all Na_2_S added to the reaction was converted into GSS^–^ below $C_{1}$. The equation of $v_{0}^{app}$ was fitted to the observed data using the non-linear least squares fit to estimate $C_{1}$.

### 4.5 Sample preparation for ESI-MS analysis

For the detection of the methylated form of glutathione persulfide, the reaction mixture was prepared as described above, and the reaction was terminated by adding 0.1% TFA. For the detection of glutathione persulfide, persulfide was derivatized with *N*-iodoacetyltyramine [48]. Glutathione persulfide was generated by mixing 5 mM GSH with serial concentrations of Na_2_S_2_ (2, 20, 200, 2000 μM) and incubating at 37 °C for 5 min. The reaction mixture was supplemented with 2 mM *N*-iodoacetyltyramine and incubated at room temperature for 1 h. After the 1-h incubation, the reaction mixture was mixed with TFA and analyzed on ESI-MS.

### 4.6 ESI-MS analysis of GSSH derivative and GSSMe

A liquid chromatograph hyphenated with a quadrupole/time-of-flight mass spectrometer was used. X500R (AB Sciex, Foster City, CA, USA) was equipped with Prominence UFLC (Shimadzu, Kyoto, Japan) as the liquid chromatograph. Acetonitrile (ACN, LC-MS grade), water (LC-MS grade), formic acid (FA, 98−100%), and 1 M ammonium formate solution were obtained from Kanto Chemical (Tokyo, Japan).

The ESI source parameters for GSSH derivative were as follows: ionspray voltage floating 5500 V; temperature 500 °C; ion source gas 1 (GS1) 50 psi; ion source gas 2 (GS2) 60 psi; curtain gas (CUR) 25 psi; declustering potential (DP) 50 V; collision energy (CE) 10 V; and range *m/z* 50–800. Chromatographic separation was accomplished with a CORTECS T3 column (100 × 2.1 mm I.D., 2.7 μm, Waters, Milford, MA) maintained at 40 °C. The mobile phase was composed of 0.1% FA and 10 mM ammonium formate (A) and ACN (B), and was eluted in the following gradient: 0 to 12 min: 10−60% B; 12 to 18 min: 60−100% B; 18 to 23 min: 100% B; and 23 to 30 min: 10% B. The flow rate was 0.3 mL/min, and the injection volume was 2 μL.

The ESI source parameters for GSSMe were as follows: ionspray voltage floating 5500 V; temperature 500 °C; GS1 50 psi; GS2 60 psi; CUR 25 psi; DP 50 V; CE 10 V; and range *m/z* 50–800. Chromatographic separation was accomplished with an Intrada Organic Acid (150 mm × 2 mm I.D., Imtakt Corp., Kyoto, Japan) maintained at 60 °C. The mobile phase was composed of 10% ACN, 90% water, and 0.1% FA (A) and 10% ACN and 90% 100 mM ammonium formate (B), and was eluted in the following gradient: 0 to 1 min: 0% B; 1 to 7 min: 0−100% B; 7 to 10 min: 100% B; and 10 to 20 min: 0% B. The flow rate was 0.2 mL/min, and the injection volume was 5 μL.

### 4.7 Quantification of RSS by fluorescence microscopy and transfection of TPMT

The effect of TPMT expression on endogenous RSS was examined using SSP4 (DOJINDO, Cat. No. SB10), an RSS-specific fluorescent dye [49]. The open reading frame of TPMT was cloned into pCI-neo vector. The open reading frame of mCherry was fused with H2B and cloned into pEF-BOS. Either the TPMT-expressing vector or the control vector was mixed with mCherry-expressing vector at a ratio of 9:1 and used for the transfection. COS-1 cells were transfected with either TPMT/pCI-neo or control vector using acidified polyethyleneimine [50]. At 24 h after transfection, the cells were washed with serum-free DMEM and stained for 15 min with SSP4 working solution consisting of DMEM, 500 μM cetyltrimethylammonium bromide, and 20 μM SSP4. The fluorescence intensity of SSP4 was quantified using Zeiss LSM700, and images were analyzed using ImageJ Fiji. Each image was divided into two categories of area: mCherry-positive and negative areas. The ratio of SSP4 signal per pixel, SSP4/pixel, was calculated in the mCherry-positive and negative areas, and the ratio of SSP4/pixel in the mCherry-positive area to that in the negative area, (SSP4/pixel_posi_)/(SSP4/pixel_nega_), was calculated as well. The (SSP4/pixel_posi_)/(SSP4/pixel_nega_) values of images were represented as a dot plot.

### 4.8 Establishment of a hepatoma line with stable knockdown TPMT gene

Hepa 1-6 cells, a mouse cell line derived from hepatoma, were obtained from RIKEN BioResource Research Center (RCB1638) and maintained with DMEM high glucose (Sigma-Aldrich, Cat. No. D5796) supplemented with 10% fetal bovine serum. A lentivirus vector, TRC2-pLKO-puro, encoding predesigned short hairpin RNA against mouse TPMT (shTPMT) (Sigma-Aldrich, MISSION, Cat. No. TRCN0000305342), was purchased from Sigma-Aldrich. The target sequence of shTPMT is 5′-CTGGTAGTGGAGCACACCTTT-3′ at 3′-UTR. pCAG-HIVgp (Cat. No. RDB04394) and pCMV-VSV-G-RSV-Rev (Cat. No. RDB04393) were provided by the RIKEN BioResource Research Center through the National BioResource Project of MEXT, Japan [51]. A control lentivirus was made with a MISSION TRC2 pLKO.5-puro vector encoding shRNA that targets no known genes from any species (Sigma-Aldrich, Cat. No. SHC216).

COS-1 cells were seeded in a 10 cm dish at a concentration of 3.0 × 10^6^ cells and transfected with 0.5 μg each of TRC2-pLKO-puro, pCAG-HIVgp, and pCMV-VSV-G-RSV-Rev vectors using acidified polyethyleneimine [50]. After 48 h, the supernatant was collected and passed through a 0.45 μm membrane filter. The lentivirus solution was concentrated using a Lenti-X concentrator (Takara Bio, Cat. No. Z1231 N). Viral RNA was isolated using NucleoSpin RNA Virus (Takara Bio, Cat. No. 740965), and virus titer was examined using real-time PCR with a primer pair amplifying the psi packaging signal: forward, 5′-CGA CTG GTG AGT ACG CCA AA-3’; reverse, 5′-CGC ACC CAT CTC TCT CCT TCT-3’. Hepa 1-6 cells were infected with the concentrated virus and incubated for 48 h. Then, the cells were selected in the presence of 0.1 μg/mL of puromycin. TPMT expression was examined using qPCR with the following primers: TPMT forward, 5′-ATC AGG AGC AAG GGC ATC AG-3’; TPMT reverse, 5′-GAG GGG GAA AAA CAC TCG CA-3’; GAPDH forward, 5′-AGG TCG GTG TGA ACG GAT TTG-3’; GAPDH reverse, 5′-TGT AGA CCA TGT AGT TGA GGT CA-3’. RNA extraction, reverse transcription, and real-time PCR were performed as described previously [10].

### 4.9 Cytotoxicity assay using RSS

Hepa 1-6 cells, which were stably infected with shTPMT-expressing or control lentivirus, were seeded in 96-well plates at 2 × 10^4^ cells per well and cultured for 24 h. The cells were exposed to the indicated concentration of Na_2_S_3_ (Dojindo, Cat. No. SB03) for an additional 24 h. Cell viability was assessed using the CellTiter 96 Aqueous One Solution (Promega, Cat. No. G3582), in accordance with the manufacturer's instructions, as previously described [10]. All samples were prepared in quadruplicate for each experiment.

### 4.10 Mice

B6D2F1 mice were purchased from CLEA Japan (Kawasaki, Japan). ICR mice were purchased from Jackson Laboratory Japan (Yokohama, Japan).

### 4.11 Generation of TPMT knockout mice

TPMT knockout mice were generated by CRISPR–Cas9 genome editing using an electroporation method partially modified from previous reports [52,53]. The target sequences of four crRNAs flanking exon 4 of the *TPMT* gene were designed as follows (TpmtL1: ACGGGGCCTTAGGCATGGCA, TpmtL2: GAGTTTTAGTTCCAGCCAGG, TpmtR1: CAGATGGGTGTCTCTACCAC and TpmtR2: ACACTGCTGCTCTCTACCAC). Pre-annealed crRNAs (Alt-R CRISPR–Cas9 crRNA, IDT)/tracrRNA (Alt-R CRISPR–Cas9 tracrRNA, IDT) (3 μM), recombinant Cas9 protein (100 ng/μl; TrueCut Cas9 Protein v2, Thermo Fisher Scientific) in Opti-MEM I (Thermo Fisher Scientific) were introduced to B6D2F1-derived zygotes by electroporation, and then edited embryos were transferred to the oviduct of pseudopregnant ICR female. KO alleles were confirmed by sequencing analysis of PCR products using the following primer sets: 5′- TCCATCATCACAGAAAATTAATCA-3′ (Tpmt-3) and 5′- ACCTTCAGAGTGCTGAGCTG -3′ (Tpmt-4).

### 4.12 ESI-MS analysis of TMS in mouse urine

For detection of TMS, 400 μL of methanol containing TMS-*d*_9_ as the internal standard was added to 100 μL urine collected from a mouse. The mixture was vortexed, and centrifuged at 10,000×*g* for 10 min. The resulting supernatant was subjected to ESI-MS for quantification of TMS.

The ESI source parameters for TMS were as follows: ionspray voltage floating 5500 V, temperature 200 °C, ion source gas 1 (GS1) 50 psi, ion source gas 2 (GS2) 60 psi, curtain gas (CUR) 25 psi, declustering potential (DP) 50 V, collision energy (CE) 10 V, and scan range *m/z* 50–200. Chromatographic separation was performed with a GS320A-2E column (250 × 2.0 mm I.D., Showa Denko, Tokyo, Japan) maintained at 40 °C. The mobile phase was consisted of 20% of 10 mM ammonium formate and 80% of MeOH. The flow rate was 0.1 mL/min, and the injection volume was 10 μL.

### 4.13 Sampling of binding conformations of ligand–enzyme complex using simulated annealing–molecular dynamics simulation

Possible binding conformations of ligands, hydrodisulfide (HSS^−^), glutathione persulfide anion (GSS^−^), and methylselenide (MeSe^−^), within the active site of the TPMT/SAM complex were examined using the simulated annealing**–**molecular dynamics (SA-MD) simulation, as previously described [54]. Given the low p*K*_a_ values [6,7,9], we selected the deprotonated anionic forms as the ligands.

The complex structures of TPMT, SAM, and ligands were processed to construct topologies and coordinates for MD simulations, as previously described [11,54]. The crystal structure of Mouse TPMT (PDB 2GB4) [17] was used as the initial structure, and side chains were protonated on the H++ server. The conformations of SAM, HSS^−^, GSS^−^, and MeSe^−^ were optimized by the restricted Hartree**–**Fock method (RHF) using the 6-31G* basis set by NWChem ver. 7.0.2 [55]. Charge distribution was calculated by the RHF method using the 6-31+G** basis set by GAMESS 2023.R2 [56]. Electrostatic potentials were evaluated at points distributed over the solvent-accessible molecular surface, defined using the Connolly algorithm, and used for the subsequent potential-derived charge fitting. Restrained electrostatic potential charge was generated using ANTECHAMBER in AmberTools22 [57], and atom types were assigned using the general AMBER force field (GAFF2). The resulting topology was validated using PARMCHK2.

The topologies of the TPMT/SAM/ligand complexes were generated using tLEaP with AMBER ff19SB force field [58] and GAFF2. A truncated octahedral simulation box was set and filled with OPC water. The minimum distance between the box wall and the solute was set at 1.2 nm. The charge was neutralized by adding Na^+^ ions. The topology and coordinate files were converted into GROMACS format using ParmEd [59]. The Lennard-Jones parameters of Se were set to σ = 0.377741 nm and ε = 1.21754 kJ/mol in the GROMACS topology file, in accordance with previous reports [[60], [61], [62]]. Other parameters were also modified to replace sulfur with Se.

MD simulations were performed using GROMACS 2024.1 [63]. Bond lengths were constrained using the LINCS method. The Verlet scheme was used as the non-bonded cut-off scheme. Cut-off was set to 1.2 nm for van der Waals and electrostatic interactions. The particle-mesh Ewald method was used to calculate long-range electrostatic interactions. A three-dimensional periodic boundary condition was applied. The geometry was optimized using the steepest descent with a threshold of 1000 kJ/mol. A leapfrog integration algorithm was applied to the following procedures. The time step was set to 2 fs for NVT and NPT equilibration, whereas it was set to 1 fs in the production MD using simulated annealing. The temperature was coupled to 300 K in NVT equilibration using the velocity rescaling or the Bussi-Donadio-Parrinello thermostat. The pressure was coupled to 100 kPa, and the C-rescale barostat and the Parrinello-Rahman barostat were used in NPT equilibration and production MD, respectively.

Possible binding conformations of the ligands within the active site of the TPMT/SAM complex were sampled using the simulated annealing method. The system was divided into two temperature-coupling groups: one group was composed of TPMT, SAM, Na^+^, and water, and the other was composed of a ligand. The first group was heated to 350 K from 0 to 500 ps, maintained at 350 K from 500 to 1000 ps, cooled to 300 K from 1000 to 2000 ps, and equilibrated at 300 K from 2000 to 2500 ps. The ligand was heated to 1000 K from 0 to 500 ps, kept at 1000 K from 500 to 1000 ps, cooled to 300 K from 1000 to 2000 ps, and equilibrated at 300 K from 2000 to 2500 ps. The cycle was repeated 50 times in a single 100-ns MD simulation (Fig. S2A). The simulation was repeated five times, and 200 conformations were collected. After the production MD, the trajectory was processed using the TRJCONV module, and averaged conformations were calculated using the RMS module from the equilibration phases (2000–2500 ps of each cycle) of the SA-MD simulation. The averaged conformations were energy-minimized and used in calculations with the FMO method, as described below.

In the conformation sampling using the simulated annealing, the position of ligands was restrained during energy minimization, NVT and NPT equilibrations, and production MD. Restraints were applied to the distance among the following atoms using a SHAKE algorithm: a sulfur atom of SAM (S_SAM_), a carbon atom of the methyl group in SAM (C_SAM_), and a sulfur atom of HSS^−^ and GSS^−^ that is deprotonated and may work as the methyl acceptor (S_1_). The S_1_–S_SAM_ and S_1_–C_SAM_ distances were set to 0.54 nm and 0.36 nm, respectively. MeSe^−^ was also restrained, and the distances between Se–C1 and Se–S3 were set to 0.37 nm and 0.55 nm, respectively.

### 4.14 Fragment Molecular Orbital method (FMO method)

Conformations sampled in the SA-MD simulation were analyzed using the FMO method [64], which is implemented in GAMESS 2023.R2 [65]. Analyses using the FMO method were performed as described previously [11,54]. The structures of TPMT/SAM and ligands were processed using Fassio 23.1.5 [66]. The residue of TPMT was divided into fragments, each containing one residue. GSS^−^ and SAM were divided into three fragments each (Fig. S2B and C).

The FMOs were calculated using the third-generation density-functional tight-binding (DFTB3) method with a 3OB-3-1 parameter set [67]. Electron dispersion was corrected using the modified third-generation empirical dispersion correction developed by Grimme et al., incorporating the Becke-Johnson dumping function, DFT-D3(BJ) [68]. The solvent effect was incorporated using the conductor-like polarizable continuum model (PCM).

The pair interaction energy (PIE) was calculated using the two-body expansion of the FMO method with DFTB3 and PCM (FMO2-DFTB3/PCM), which generates the matrix of *PIE*_*i*_,_*j*_, where *i* and *j* represent the FMOs of the TPMT/SAM complex and the ligands (Fig. S2B and D). PIEs between the ligand and the TPMT/SAM complex were summed for each residue of the TPMT/SAM complex as $\sum_{j}{PIE}_{i,j}$, which represents the interaction energy between the ligand (*j*) and each fragment from the TPMT/SAM complex (*i*). PIEs were also summed for all the pairs of ligands and fragments from the TPMT/SAM complex as $\sum_{j}\sum_{i}{PIE}_{i,j}$, which represents the interaction energy between the ligand and the TPMT/SAM complex. PIE decomposition analysis was described previously [69].

## 5 Identification of stable binding conformations

The PIEs of the conformations sampled in the SA-MD simulation were analyzed to identify stable binding conformations of ligands within the active site of the TPMT/SAM complex, as previously described [54]. The matrices of the PIEs were analyzed by agglomerative clustering using the AgglomerativeClustering class from the cluster module in scikit-learn 1.5.1. Dendrograms were generated using the hierarchical clustering functions in the clustering package in SciPy 1.13.1. Clusters were defined on the basis of the dendrogram. The sums of PIEs, $\sum_{j}\sum_{i}{PIE}_{i,j}$, were plotted for each conformation within the cluster to identify the most stable cluster. The results were depicted as dot and box-whisker plots using Matplotlib and seaborn. The sums of PIEs for the clusters were also analyzed using the Tukey–Kramer test with statmodels 0.14.2. We identified a stable cluster that exhibited the strongest interaction energy, and this energy was statistically significantly different from that of the other clusters.

In the analysis of HSS^−^, a total of 200 conformations were sampled from SA-MD simulations and grouped into nine clusters (Fig. S2E). The sums of PIEs for each cluster are shown in Fig. 4B, and the results of the Tukey–Kramer test are presented in Fig. S2F.

In the analysis of GSS^−^, two different conformations were used as starting models. For each conformation, ten independent SA-MD simulations were performed. From each set of simulations, 400 conformations were sampled and grouped into clusters (Figs. S3A and S4A). The sums of PIEs were depicted as dot plots (Figs. S3B and S4B) and analyzed using the Tukey–Kramer test (Figs. S3C and S4C). Stable conformations were identified in two samplings derived from different starting models.

In the analysis of MeSe^−^, the clustering results are shown in Fig. S6A, and the corresponding sums of PIEs are presented in Fig. 6D. The results of the Tukey–Kramer test are shown in Fig. S6B.

### 5.1 Calculation of binding energy of ligand to TPMT/SAM complex

The most stable conformations in the selected clusters were geometry-optimized, and the binding energy of the ligand was calculated. The geometry of the TPMT/SAM/ligand complex was optimized using the FMO2-DFTB3/PCM method for more than 1000 steps. The binding energy of a ligand to the TPMT/SAM complex was calculated using the three-body expansion of the FMO method with DFTB3 and PCM (FMO3-DFTB3/PCM). The dispersion-corrected free energies of the TPMT/SAM/ligand complex (*E*_complex_), the TPMT/SAM complex (*E*_receptor_), and a ligand (*E*_ligand_) were calculated using the FMO3-DFTB3/PCM method. The final binding energy (*ΔG*_bind_) was calculated as *ΔG*_bind_ = *E*_complex_ – (*E*_receptor_ + *E*_ligand_).

### 5.2 Conventional molecular dynamics simulation evaluating the stability of ligands within the active site of TPMT

The dynamics of ligands within the active site of TPMT were analyzed by conventional MD simulations using the best conformations in the stable clusters as the initial conformation. Three conformations, two of HSS^−^ (clusters 0 and 6 in Fig. 4) and one of MeSe^–^ (cluster 3 in Fig. 6), were used as initial structures. The MD simulation was performed using GROMACS under the same conditions as those described for the SA-MD simulation. However, the temperature was maintained at 310 K, and restraints on the distance between the ligands and SAM were not applied. Production MD was performed for more than 20 ns. In the HSS^−^ molecule, deprotonated anionic sulfur and protonated sulfur were designated as S_1_ and S_2_, respectively. The DISTANCE module was used to calculate the distance between S_1_ or S_2_ of HSS^−^ and a carbon of the methyl group of SAM (C_SAM_). The ANGLE module was used to calculate angle *θ* formed by a sulfur of SAM (S_SAM_), C_SAM_, and either S_1_, S_2_, or Se (Fig. 8D–F).

### 5.3 Quantum calculation of HOMO energy levels of methyl acceptors

HSS^−^, HS^−^, HSe^−^, and MeSe^−^ were complexed with monomethylated guanidine (Fig. 7A–D), and the geometry was optimized using the DRIVER module of NWChem with MP2/aug-cc-pVDZ until convergence with the default convergence criteria. Vibrational analysis showed that all normal modes had positive frequencies. The first ionization energy was estimated using the ΔSCF (delta self-consistent field) method by calculating the total electronic energies of the closed-shell neutral species and the corresponding open-shell cation, obtained by removing one electron from the neutral molecule. The difference between these total energies was used to approximate the ionization energy corresponding to the HOMO energy level. Total energy calculation was performed using NWChem with MP2/aug-cc-pVDZ or GAMESS with ωB97X-D/aug-cc-pVTZ. The resulting structure and orbitals were visualized using wxMacMolPlt.

## Funding

This work was supported in part by Grants-in-Aid for Scientific Research from the Ministry of Education, Culture, Sports, Science and Technology of Japan [24K09793 (Y. F.), 24H00749 (Y. O.), 24K21304 (Y. O.), 25K02898 (Y. Y.), 22K05345 (N. S.)]. Additional support was provided by the ACT-UR Grant Program (#4366, #4489) from Agilent Technologies. This research was also supported by Research Support Project for Life Science and Drug Discovery [Basis for Supporting Innovative Drug Discovery and Life Science Research (BINDS)] from AMED under Grant Number JP25ama121049 to I.H.

## CRediT authorship contribution statement

**Yasunori Fukumoto:** Conceptualization, Data curation, Formal analysis, Funding acquisition, Investigation, Methodology, Project administration, Software, Supervision, Validation, Visualization, Writing – original draft, Writing – review & editing. **Momoka Uchida:** Data curation, Investigation, Validation. **Yoshikazu Yamagishi:** Data curation, Funding acquisition, Investigation, Methodology, Supervision, Writing – review & editing. **Natsu Saito:** Data curation, Investigation, Validation. **Ayune Watanabe:** Data curation, Investigation, Resources, Validation. **Rina Mukasa:** Data curation, Investigation, Validation. **Ryosuke Kobayashi:** Resources. **Takuro Horii:** Resources. **Izuho Hatada:** Funding acquisition, Resources. **Yu-ki Tanaka:** Funding acquisition, Investigation, Methodology, Supervision. **Noriyuki Suzuki:** Funding acquisition, Supervision, Writing – review & editing. **Yasumitsu Ogra:** Funding acquisition, Project administration, Supervision, Writing – review & editing.

## Declaration of competing interest

The authors declare that they have no known competing financial interests or personal relationships that could have appeared to influence the work reported in this paper.

## Data Availability

No data was used for the research described in the article.

## Abbreviations

RSS, reactive sulfur species; GSH, glutathione; GSS^−^, glutathione persulfide; GSSR, *N*-acetyltyramine conjugate of GSS^−^; DMDSe, dimethyldiselenide; MeSe^−^, methylselenide anion; GSSe^–^, glutathione perselenide; TMSe, trimethylselenonium ion; TMS, trimethylsulfonium ion; TPMT, thiopurine *S*-methyltransferase; INMT, indolethylamine *N*-methyltransferase; SQOR, sulfide quinone oxidoreductase; ETHE1, persulfide dioxygenase; SAH, *S*-adenosylhomocysteine; SA-MD, simulated annealing–molecular dynamics; RMSD, root-mean-square deviation; FMO, fragment molecular orbital; DFTB3, third-generation density-functional tight-binding; PCM, polarizable continuum model; RHF, restricted Hartree–Fock method; HOMO, highest occupied molecular orbital.
