# Supplementary material for: Reactive sulfur species are inactivated and excreted as trimethylsulfonium ion by thiopurine S-methyltransferase
Source: Redox Biol. 2026 Apr 1;93:104144. doi: 10.1016/j.redox.2026.104144 (PMC13089183; doi:10.1016/j.redox.2026.104144)
Supplement: Multimedia component 1 [file mmc1.docx]

**Supplementary Results**

**Supplementary Result 1: Kinetic modeling of GSH-enhanced TPMT-mediated methylation of H_2_S.**

To investigate how GSH enhances the TPMT-mediated methylation of H_2_S, we hypothesized that the reaction proceeds through two distinct substrates and mechanisms: direct methylation of H_2_S and methylation of GSS^−^ formed via the GSH-dependent oxidation of H_2_S. To test this hypothesis, the observed data were modeled using a linear combination of Michaelis–Menten equations for GSS^−^ and H_2_S, applying the respective *K*_m_ and *V*_max_ values derived from the GSH/Na_2_S_2_ and Na_2_S reactions (Figure 2F). The first pathway involves GSS^−^, which is methylated by TPMT at an approximately 700-fold higher efficiency than H_2_S (Figure 2I). However, the formation of GSS^−^ depends on the oxidative conversion of H_2_S, which is limited at high Na_2_S concentrations. The second pathway is the direct, slower methylation of H_2_S itself, which persists even at high substrate concentrations. These two mechanisms were each modeled with Michaelis–Menten kinetics and then combined to fit the observed reaction data. Details are provided in the *Linear combination of two Michaelis–Menten equations* section in Methods. For comparison, the same data were also fitted using a standard Michaelis–Menten model (Figure S1B).

The linear combination model provided a better fit, as indicated by a lower sum of squared residuals (16.4) than that of the standard model (21.2). The linear combination model yielded *K*_m_ = 1297 μM, *V*_max_ = 6.47 nmol/min·mg, and *V*_max_/*K*_m_ = 0.0050 L/min·g (Figure 2I). In contrast, the standard model gave *K*_m_ = 2.50 μM, *V*_max_ = 5.57 nmol/min·mg, and *V*_max_/*K*_m_ = 2.2 L/min·g (Figure S1C). The *V*_max_ values were similar, but a major difference was observed in *K*_m_. This discrepancy reflects the inability of the standard model to account for the increased initial velocity *V*_0_ at higher Na_2_S concentrations (Figure 2F), which is more accurately captured by the linear combination model (Figure S1B). These modeling results support the hypothesis that GSH enhances the TPMT-mediated methylation of H_2_S primarily by promoting the formation of GSS^−^, which serves as a kinetically preferred substrate for this reaction.

**Supplementary Result 2: Molecular dynamics simulations reveal ligand dynamics within the TPMT active site**

To investigate the dynamic behavior of ligands within the TPMT active site, we performed conventional MD simulations by removing the distance restraints between SAM and HSS^−^. The most stable conformations from clusters 0 and 6 in Figure 4 were selected as initial structures, as these clusters represented distinct binding conformations of HSS^−^ (Figures 4D, G). In both simulations, HSS^−^ was initially stabilized within the active site but eventually dissociated over the course of the trajectory (Figures 8A, B, S7). Notably, the dynamics of HSS^−^ differed significantly between clusters 0 and 6. In the cluster 6-based simulation, the protonated sulfur atom (S_2_) remained consistently associated with SAM, whereas the deprotonated sulfur (S_1_) stayed farther from the C_SAM_ atom (Figure 8A, blue and red lines). Among 20,000 frames extracted from the initial 20 ns of the trajectory, S_1_ and S_2_ were located within 4 Å of C_SAM_ in 6.6% and 25.5% of frames, respectively (Table S3). In contrast, cluster 0-based simulation showed frequent alterations in position between S_1_ and S_2_, reflected in the fluctuating distances between C_SAM_ and each sulfur atom (Figure 8B, green line). In this case, S_1_ and S_2_ were within 4 Å of C_SAM_ in 35.6% and 29.3% of frames, respectively (Table S3). The dynamics of MeSe^−^ were also examined using cluster 3 in Figure 6 as the initial structure. Unlike HSS^−^, the distance between Se and SAM was constant during the MD simulation (Figures 8C, S8), suggesting a stable association of MeSe^−^ with the active site of TPMT. Se was within 4 Å of C_SAM_ in 54.7% of the frames. Taken together, these results indicate that sulfur and Se atoms are capable of associating with the active center, even in the absence of distance restraints.

The *θ* angle formed by S_SAM_, C_SAM_, and a third atom (S_1_, S_2_, or Se) was examined as a geometric parameter indicative of geometric readiness for an SN2 reaction among the frames where the S–C_SAM_ distance was within 4 Å (Figures 8D–F). In cluster 6 of HSS^−^, S_1_ and S_2_ occupied an angular range of 150° to 180° in 21.2% and 32.2% of the analyzed frames, respectively (Table S3). In cluster 0, S_1_ and S_2_ occupied the favorable angular range in 48.8% and 54.5% of frames, respectively. In the case of MeSe^−^, Se occupied the favorable angular range in 50.6% of the frames. The selected frames satisfied the geometric criteria, namely, an S/Se–C_SAM_ distance of less than 4 Å and an angle *θ* between 150° and 180°, and their spatial arrangements were conducive to nucleophilic attack. These frames were subsequently used in the analysis of the angle *φ* defined by S_1_–S_2_–C_SAM_, S_2_–S_1_–C_SAM_, and C_methyl_–Se–C_SAM_ (Figures 8D–F).

**Supplementary Discussions**

**Supplementary Discussion 1. TPMT is the primary methyltransferase for H_2_S metabolism *in vivo*.**

Our data suggest that TPMT methylates HS^−^ but not its protonated form, H_2_S. TPMT efficiently produced SAH at pH 7.7 (Figure 2H), indicating methylation of HS^−^. In contrast, the methylation was ineffective at pH 6.8 (Figure 2E), and the *V*_max_/*K*_m_ value was sufficiently low, suggesting that TPMT is unlikely to contribute significantly to H_2_S methylation under physiological conditions.

A previous study reported the *in vitro* methylation of H_2_S by METTL7B. However, only *K*_m_ was determined, and its catalytic efficiency remained unknown. METTL7B is known to act on neutral, protonated thiols (33). As the cytosolic and mitochondrial pH ranges are approximately 7.1 to 7.3 and 7.9 to 8.0, respectively (44,45), H_2_S likely exists predominantly as HS^−^ *in vivo*. Although METTL7B may contribute to H_2_S methylation under certain conditions, TPMT is more likely to be the primary methyltransferase responsible for H_2_S metabolism *in vivo*, acting on its deprotonated form, HS^−^. We therefore propose that TPMT is the primary methyltransferase for RSS, including HS^−^.

**Supplementary Discussion 2. GSH enhances substrate recognition by TPMT in the methylation of Se compounds.**

Our data suggest that GSH facilitates the recognition of both selenide and sulfide anions by TPMT at the active site. In our previous studies on Se methylation, we proposed HSe^–^ and GSSe^−^ as candidate substrates for the initial methylation step. However, the most suitable substrate remained undetermined (10). By analogy with sulfur metabolism, TPMT is likely to recognize both HSe^−^ and GSSe^–^. The *V*_max_/*K*_m_ value of the reaction involving GSH and selenite was comparable to that of GSH and Na_2_S_2_ (Figures 2I, 6C), indicating similar catalytic efficiency toward RSS and Se. Given that the methylation of GSS^–^ is more efficient than that of HS^−^ (Figure 2I), we propose that GSSe^−^ is also methylated more efficiently than HSe^−^. These results suggest that GSH enhances substrate recognition by TPMT in the Se methylation as well.

**Supplementary Figures and Legends**


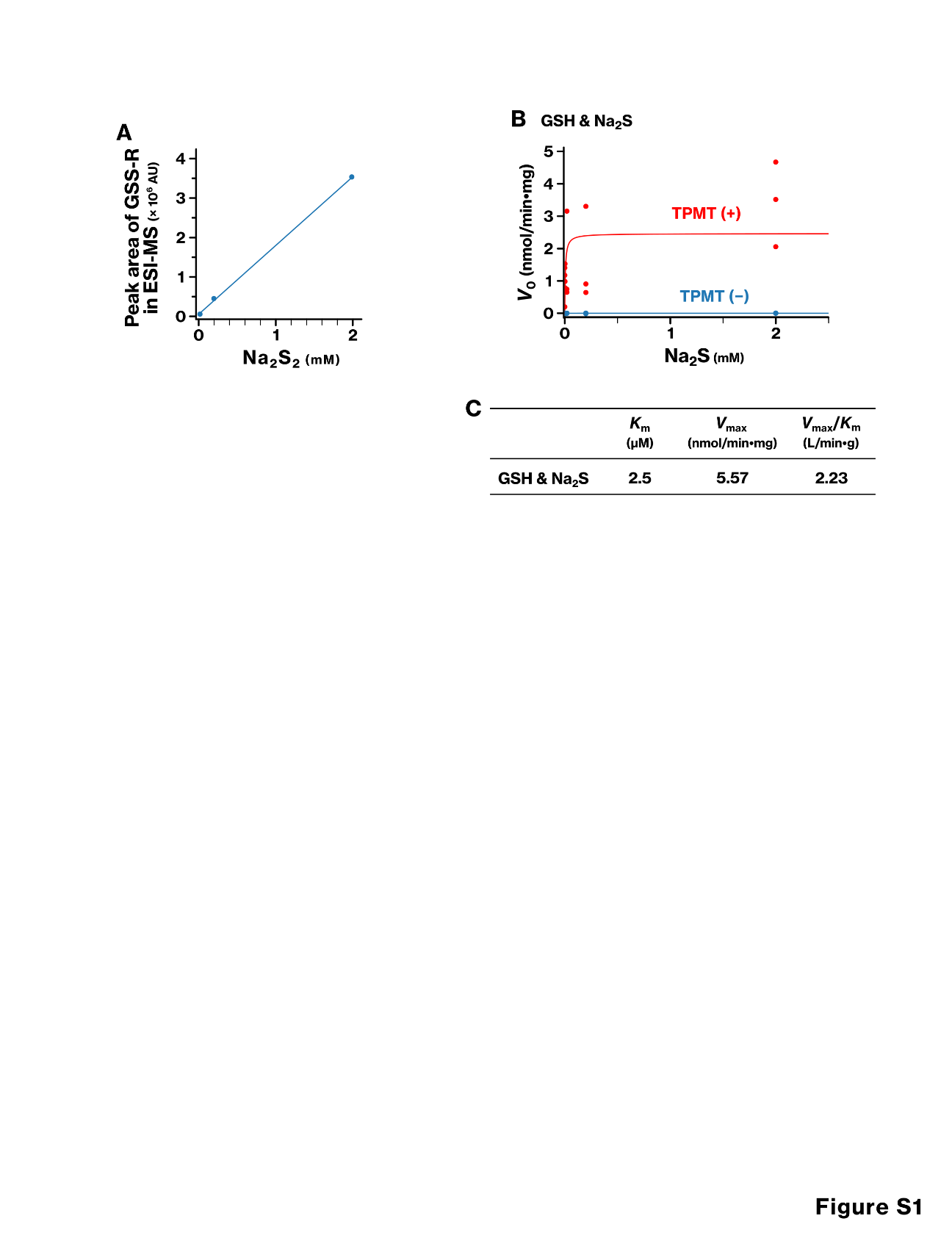


**Figure S1. ESI-MS analysis and MTase-Glo assay.**

**(A)** The same experiment as in Figure 2J, except that 2000, 200, 20, or 2 μM of Na_2_S_2_ was mixed with GSH. GSS^−^ was derivatized using *N*-iodoacetyltyramine. The peak area of the derivatized GSSH is plotted against the Na_2_S_2_ concentration in the reaction. **(B, C)** The data shown in Figure 2F were analyzed using the single-component Michaelis–Menten equation (i.e., not a linear combination), and *K*_m_ and *V*_max_ values were estimated.

**
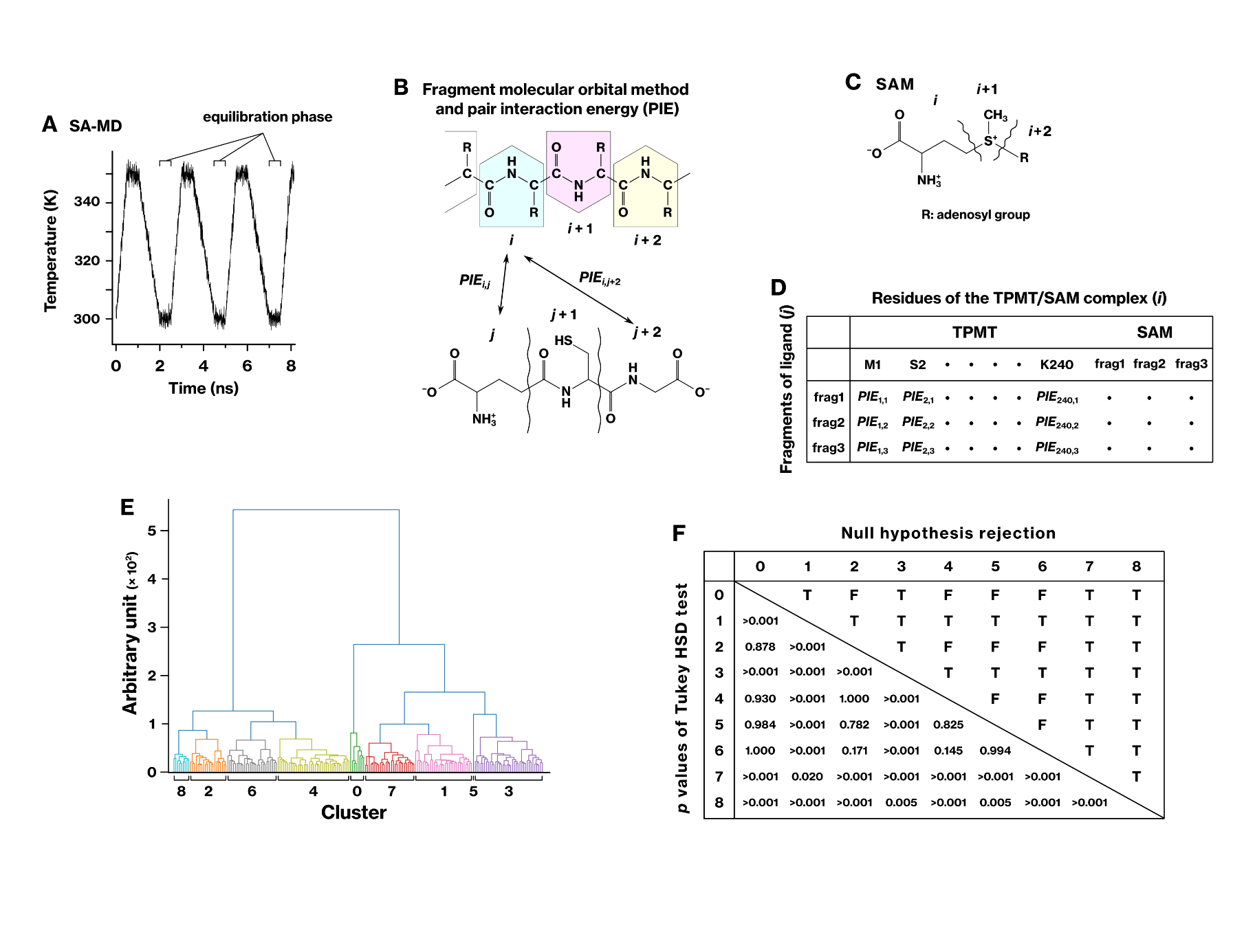
**

**Figure S2. SA-MD simulation, FMO method, and PIE analysis, predicting the conformation of the TPMT/SAM/HSS^–^ complex.**

**(A)** The binding conformation of HSS^−^ within the active site of the TPMT/SAM complex was predicted in accordance with the procedure in Figure 4A. Conformation sampling was performed using SA-MD simulation as described in Methods. The temperature profile of the TPMT/SAM complex is shown. Averaged conformation was calculated from each equilibration phase at 300 K. **(B, C, D)** PIEs were calculated for FMOs using the FMO2-DFTB3/PCM method (B). GSH (B) and SAM (C) were calculated as three fragments. A matrix of *PIE*_i,j_ was generated on the basis of each averaged conformation. **(E)** The matrices of PIEs were analyzed using agglomerative clustering and grouped into clusters. The sums of PIEs, $\sum_{j} \sum_{i} {PIE}_{i,j}$, are plotted in Figure 4B. **(F)** The PIEs for each cluster were compared with those for the other clusters using the Tukey–Kramer test. True (T) denotes a pair of clusters that differ significantly with a *p*-value of less than 0.05. F, false.


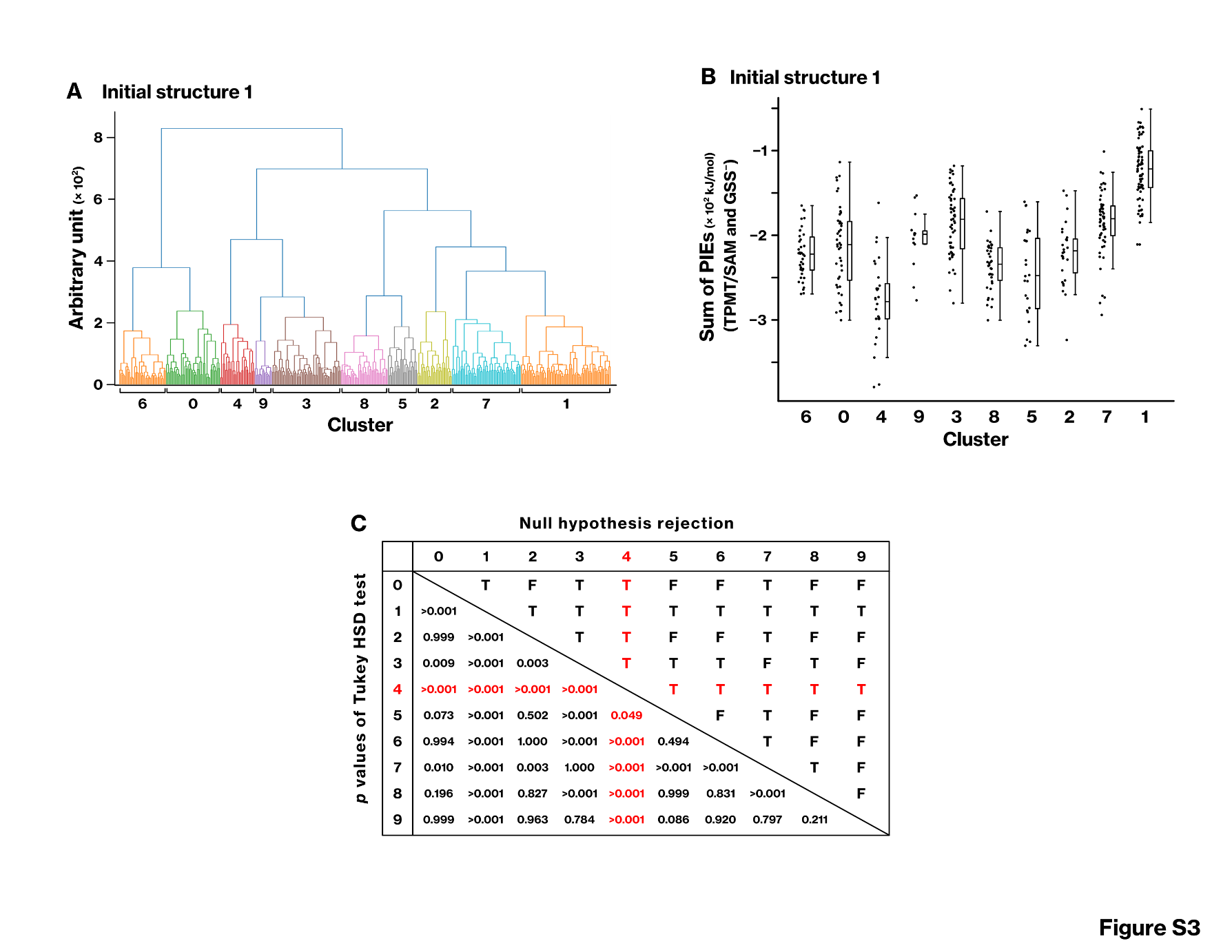


**Figure S3. SA-MD simulation, FMO method, and PIE analysis of GSS^–^ and the TPMT/SAM complex.**

**(A)** The binding conformation of GSS^–^ within the active site of the TPMT/SAM complex was predicted using the same procedure as that shown in Figures 4A and S2. Conformations were sampled using SA-MD simulation, and PIEs were calculated using the FMO2-DFTB3/PCM method. The conformations were grouped into clusters on the basis of the matrices of PIEs. **(B, C)** The sums of PIEs were plotted for each cluster, and a stable cluster was identified. The statistical significance of the difference was examined among clusters using the Tukey–Kramer test with a threshold of *p*-value of 0.05. T, true. F, false.


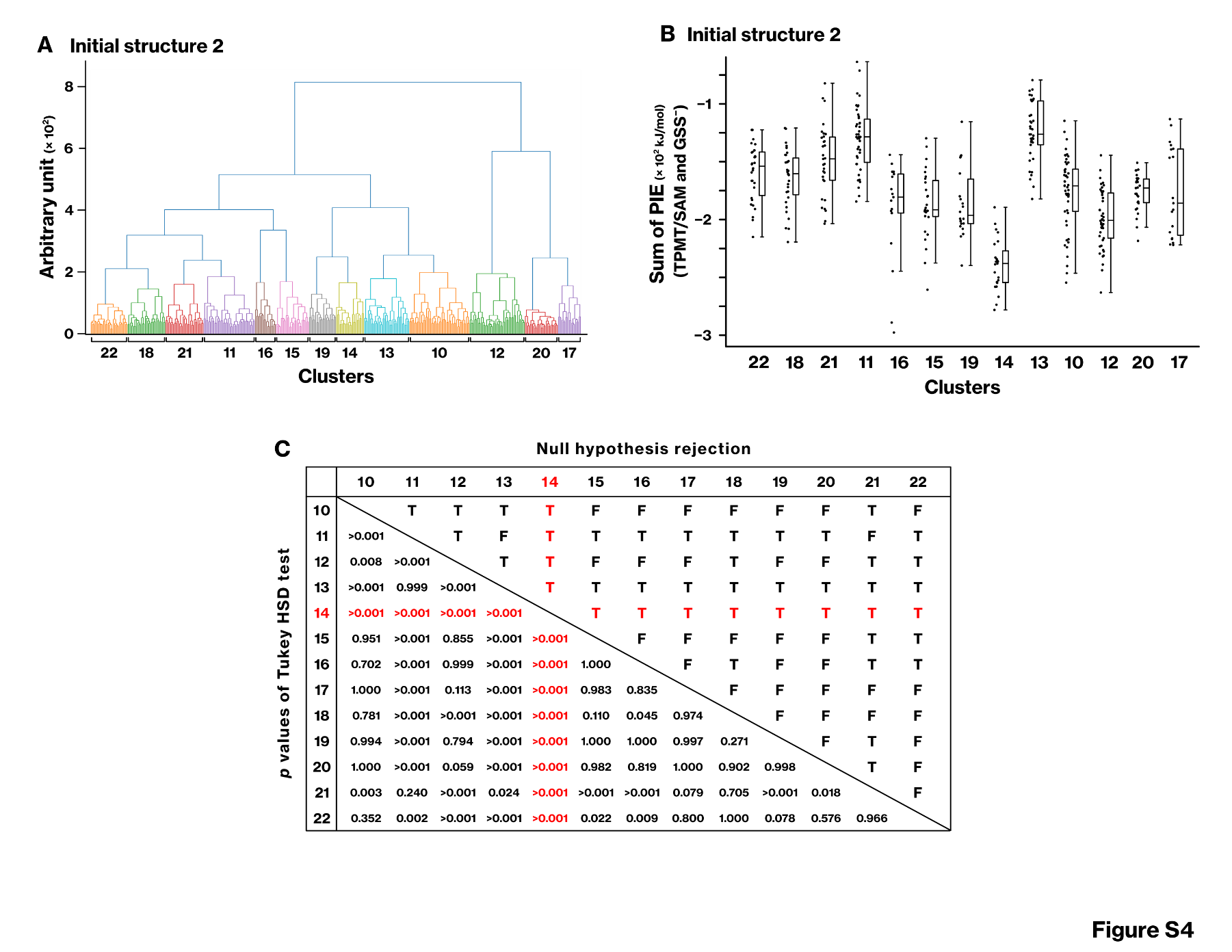


**Figure S4. SA-MD simulation, FMO method, and PIE analysis of GSS^–^ and the TPMT/SAM complex.**

**(A)** The binding conformation of GSS^–^ within the active site of the TPMT/SAM complex was predicted using a starting structure different from that used in Figure S3. The sampled conformations were clustered in accordance with the PIEs. **(B, C)** The sums of PIEs were analyzed to identify a stable cluster in the same manner as in Figure S3. T, true. F, false.


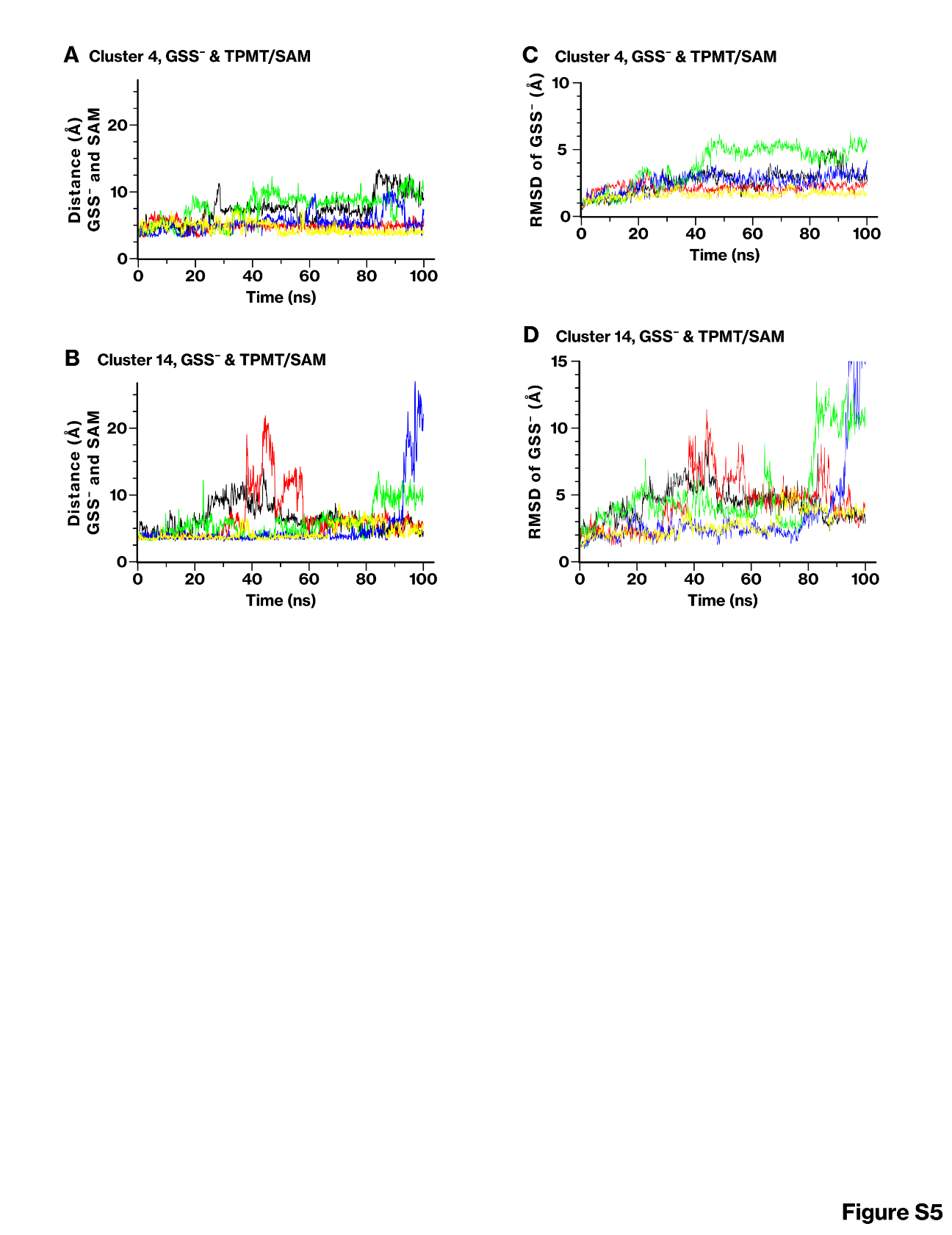


**Figure S5. Association of GSS^–^ with the active site of TPMT in conventional MD simulation.**

**(A–D)** The restraint between GSS^−^ and SAM was removed, and the dynamics of GSS^−^ within the active site of the TPMT/SAM complex were investigated using conventional MD simulation at a constant temperature of 310 K. Five repeats of MD simulations were performed independently using clusters 4 (A, C) and 14 (B, D) in Figure 4 as the starting structure. The distance between the sulfide anion in GSS^−^ and the methyl group of SAM is depicted in panels A and B. RMSD of GSS^−^ was calculated and depicted in panels C and D.


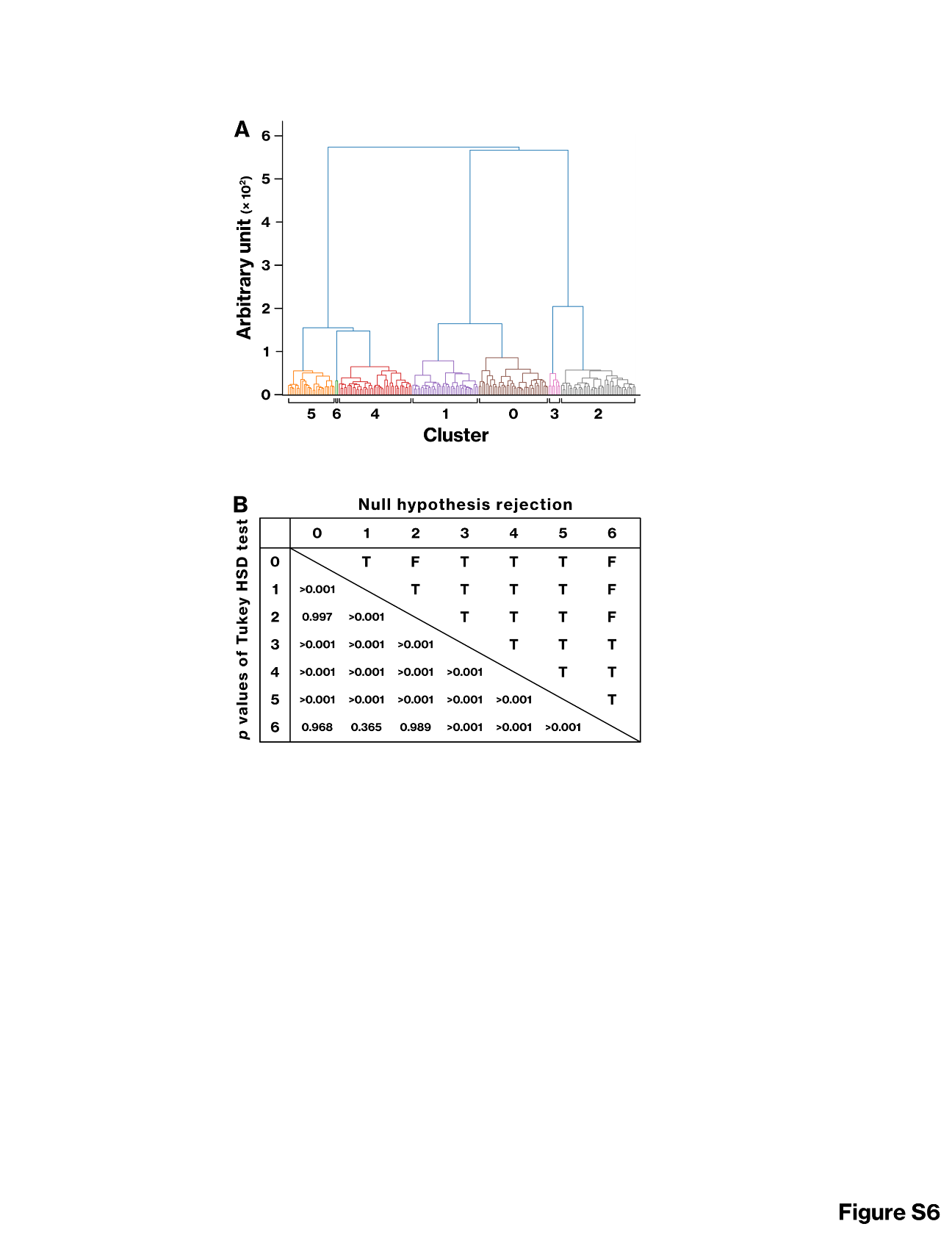


**Figure S6. SA-MD simulation, FMO method, and PIE analysis of MeSe^–^ and the TPMT/SAM complex.**

**(A)** The binding conformation of MeSe^–^ within the active site of the TPMT/SAM complex was predicted using the same procedure as that shown in Figures 3A and S2. The sampled conformations were clustered in accordance with the PIEs. The resulting dot plot is shown in Figure 6D. **(B)** The sums of PIEs were analyzed to identify a stable cluster. T, true. F, false.


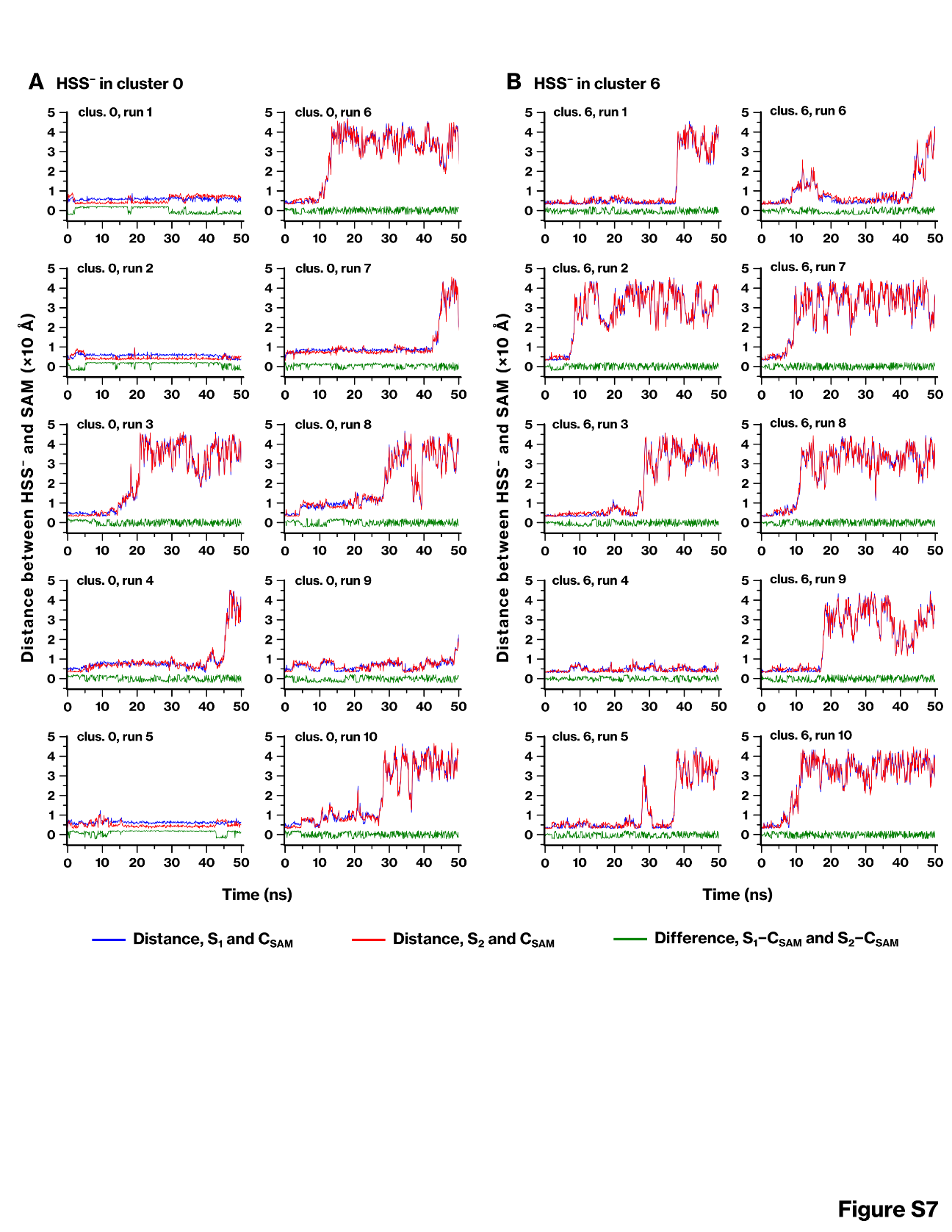


**Figure S7. Dynamics of HSS^–^ within the active site of TPMT.**

**(A, B)** MD simulations in Figures 8A and 8B are shown on a full timescale. The distance restraint between SAM and HSS^−^ was removed, and the dynamics of HSS^−^ within the active site of the TPMT/SAM complex were investigated using normal MD simulation at a constant temperature of 310 K. Ten repeats of MD simulations were performed independently using clusters 0 (A) and 6 (B) in Figure 3 as the starting structure.


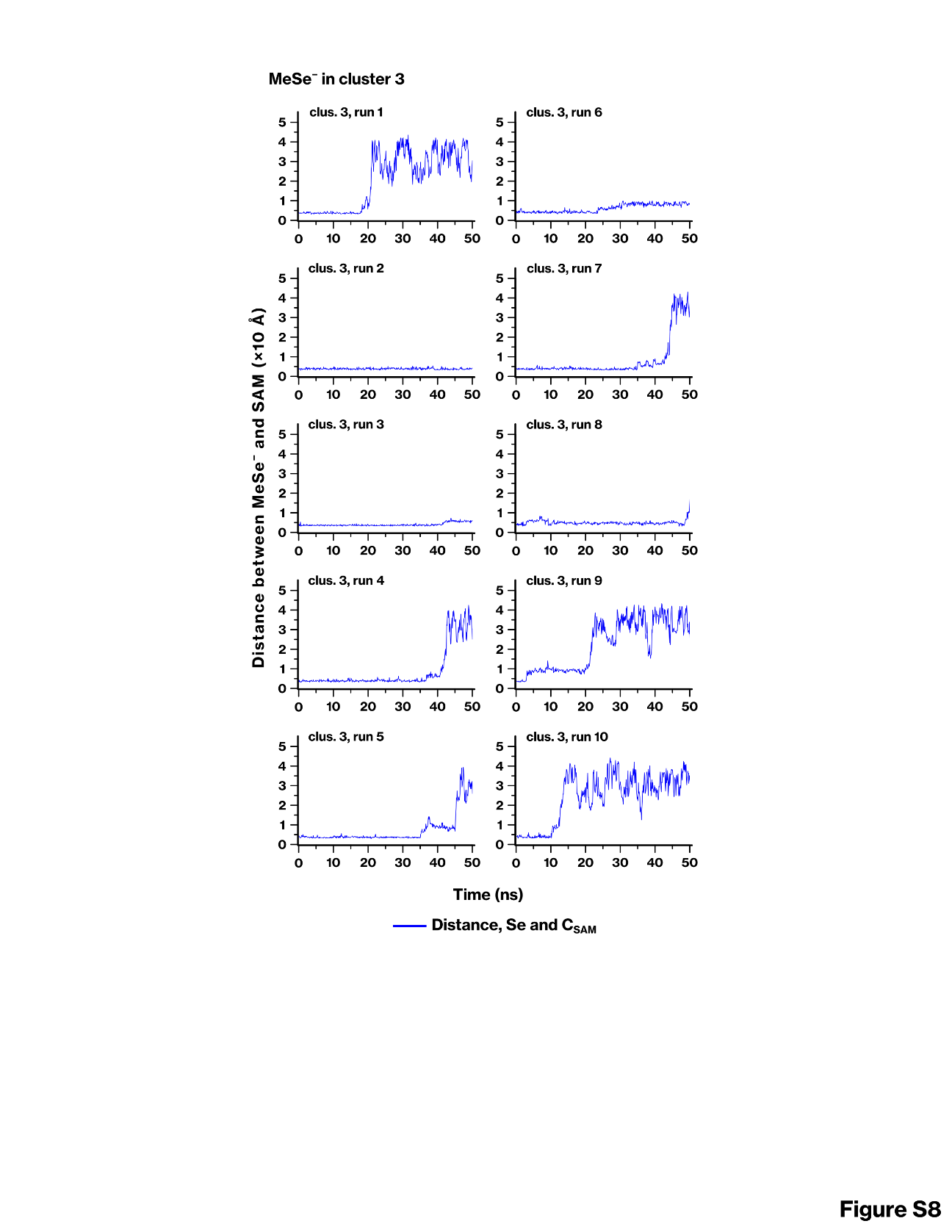


**Figure S8. Dynamics of HSe^–^ within the active site of TPMT.**

The same MD simulation in Figure 8C is shown on a full timescale. The restraint between SAM and MeSe^−^ was removed, and the dynamics of MeSe^−^ was investigated using a conventional MD simulation at 310 K. The MD simulation was repeated 10 times using cluster 3 in Figure 6.


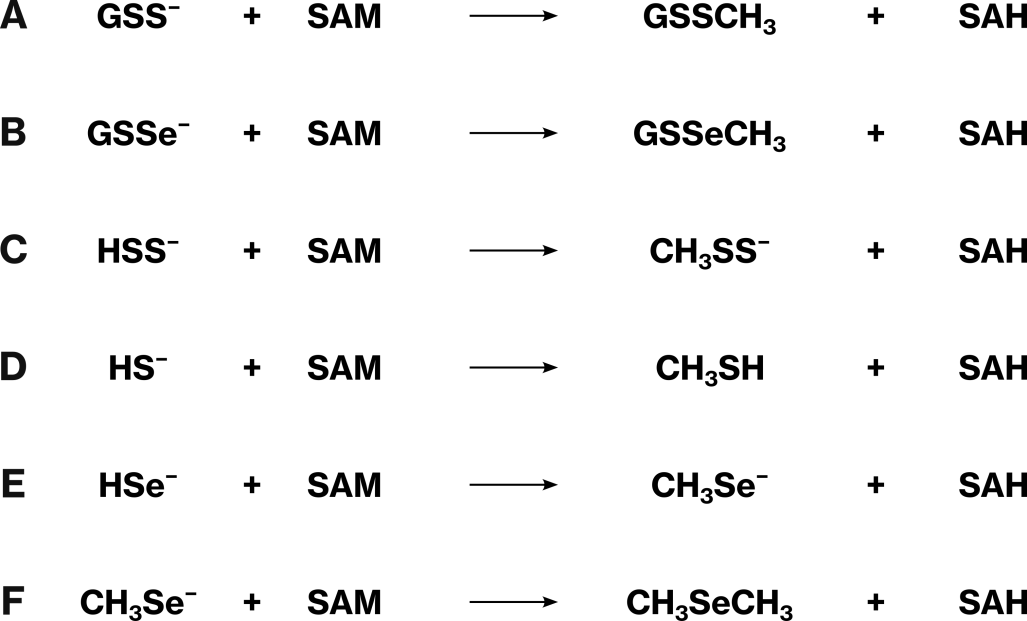


**Figure S9. Summary of TPMT-catalyzed methylation reactions.**

TPMT transfers a methyl group from SAM to reactive sulfur and selenium species, forming methylated intermediates that can be further methylated by INMT to generate fully methylated products. This scheme represents a conceptual model.

**Supplementary Tables**

**Table S1.** Assignment of precursor and product ions of methylated glutathione persulfide.

| Peak label | Elemental composition | *m/z*  theoretical | *m/z*  measured | *Δm/z*  (ppm) |
| --- | --- | --- | --- | --- |
| [M+H]^+^ | C_11_H_20_N_3_O_6_S_2_ | 354.0793 | 354.0793 | 0 |
| f1 | C_9_H_15_N_2_O_4_S_2_ | 279.0473 | 279.0473 | 0 |
| f2 | C_6_H_13_N_2_O_3_S_2_ | 225.0368 | 225.0368 | 0 |
| f3 | C_6_H_10_NO_3_S_2_ | 208.0102 | 208.0103 | 0.48 |
| f4 | C_5_H_9_N_2_OS_2_ | 177.0156 | 177.0337 | 102 |
| f5 | C_5_H_8_NO_3_ | 130.0504 | 130.0503 | −0.77 |
| f6 | C_3_H_8_NS_2_ | 122.0098 | 122.0097 | −0.82 |

Fragment ions detected in the MS/MS spectrum in Figure 2M were analyzed to assign their elemental compositions based on exact mass calculations. The table contains the elemental composition, the calculated monoisotopic *m/z*, the experimentally measured *m/z*, and the differences between theoretical and observed *m/z* values, reported in ppm.

**Table S2.**

|  | *PIE* | *E*es | *E*ct | *E*dsip | *G*sol |
| --- | --- | --- | --- | --- | --- |
| **Cluster 0** |  |  |  |  |  |
| R147 | −85 ± 4 | −99 ± 3 | −4 ± 2 | −3 ± 0 | 12 ± 4 |
| R221 | −32 ± 21 | −48 ± 22 | 0 ± 1 | 0 ± 1 | 16 ± 3 |
| SAM | −63 ± 6 | −74 ± 4 | 0 ± 0 | −1 ± 0 | 12 ± 3 |
| **Cluster 6** |  |  |  |  |  |
| R147 | −33 ± 5 | −47 ± 5 | 0 ± 0 | 0 ± 0 | 14 ± 3 |
| R221 | −77 ± 4 | −102 ± 5 | −4 ± 1 | −2 ± 0 | 24 ± 2 |
| SAM | −61 ± 5 | −76 ± 3 | 0 ± 0 | −1 ± 0 | 16 ± 4 |

The interaction between HSS^–^ and each TPMT residue shown in Figure 3C was further analyzed using pair interaction energy decomposition analysis, which revealed contributions from electrostatic interaction (*E*es), charge transfer (*E*ct), electron dispersion (*E*dsip), and solvation (*G*sol). Energies are presented as mean ± standard deviation, with units in kJ/mol.

**Table S3.** Dynamics of HSS^–^ and MeSe^–^ in the MD simulation.

|  | HSS^−^ | | HSS^−^ | | | MeSe^−^ | |  |
| --- | --- | --- | --- | --- | --- | --- | --- | --- |
|  | cluster 6 | | cluster 0 | | | cluster 3 | |  |
|  | S_1_ | S_2_ | S_1_ | S_2_ | Se | |  |  |
| S/Se–C distance within 4 Å | 6.6% | 25.5% | 35.6% | 29.3% | 54.7% | | | |
| Angle *θ* within 150°–180° | 21.2% | 32.2% | 48.8% | 54.5% | 50.6% | |  |  |
| Angle *φ* within 100°–180° | 76.5% | 54.3% | 31.5% | 9.9% | 16.5% | |  |  |

The dynamics of HSS^–^ and MeSe^−^ within the TPMT active site were analyzed over the initial 20 ns of the conventional MD simulation shown in Figures 8A–C, S7, and S8. A total of 20,000 frames were extracted from ten independent trajectories, and frames in which either S_1_, S_2_, or Se was located within 4 Å of C_SAM_ were selected for further analysis. The percentage of the selected frames is shown in the top row. In these frames, the angle formed by S_SAM_−C_SAM_ and either S_1_, S_2_, or Se was calculated and denoted as *θ* (Figures 8D–F). Frames where *θ* ranged from 150° to 180° were counted and expressed as percentage of the number of frames with the S/Se−C_SAM_ distance within 4 Å. These frames were used for the subsequent analysis of angle *φ*.
